# Clinically relevant CHK1 inhibitors abrogate wild-type and Y537S mutant ERα expression and proliferation in luminal primary and metastatic breast cancer cells

**DOI:** 10.1186/s13046-022-02360-y

**Published:** 2022-04-13

**Authors:** Sara Pescatori, Stefano Leone, Manuela Cipolletti, Stefania Bartoloni, Alessandra di Masi, Filippo Acconcia

**Affiliations:** Department of Sciences, Section Biomedical Sciences, and Technology, University Roma Tre, Viale Guglielmo Marconi, 446, I-00146 Rome, Italy

**Keywords:** Breast Cancer, Estrogen Receptor α, 17β-estradiol, 4OH-tamoxifen, CHK1, CHK2, AZD7762, Prexasertib, MK8776, Replication stress

## Abstract

**Background:**

Challenges exist in the clinical treatment of luminal estrogen receptor α (ERα)-positive breast cancers (BCs) both to prevent resistance to endocrine therapy (ET) and to treat ET-resistant metastatic BCs (MBC). Therefore, we evaluated if kinases could be new targets for the treatment of luminal primary and MBCs.

**Methods:**

~ 170 kinase inhibitors were applied to MCF-7 cells either with adaptative or genetic resistance to ET drugs and both ERα levels and cell proliferation were measured. Robust-*Z*-score calculation identified AZD7762 (CHK1/CHK2 inhibitor) as a positive hit. Subsequently, Kaplan–Meier analyses of CHK1 and CHK2 impact on ERα-positive BC patients relapse-free-survival (RFS), bioinformatic evaluations of CHK1 and CHK2 expression and activation status as a function of ERα activation status as well as drug sensitivity studies in ERα-positive BC cell lines, validation of the impact of the ATR:CHK1 and ATM:CHK2 pathways on the control of ERα stability and BC cell proliferation via inhibitor- and siRNA-based approaches, identification of the molecular mechanism required for inhibitor-dependent ERα degradation in BC and the impact of CHK1 and CHK2 inhibition on the 17β-estradiol (E2):ERα signaling, synergy proliferation studies between ET-drugs and clinically relevant CHK1 inhibitors in different luminal BC cell lines, were performed.

**Results:**

A reduced CHK1 expression correlates with a longer RFS in women with ERα-positive BCs. Interestingly, women carrying luminal A BC display an extended RFS when expressing low CHK1 levels. Accordingly, CHK1 and ERα activations are correlated in ERα-positive BC cell lines, and the ATR:CHK1 pathway controls ERα stability and cell proliferation in luminal A BC cells. Mechanistically, the generation of DNA replication stress rather than DNA damage induced by ATR:CHK1 pathway inhibition is a prerequisite for ERα degradation. Furthermore, CHK1 inhibition interferes with E2:ERα signaling to cell proliferation, and drugs approved for clinical treatment of primary and MBC (4OH-tamoxifen and the CDK4/CDK6 inhibitors abemaciclib and palbociclib) exert synergic effects with the CHK1 inhibitors in clinical trials for the treatment of solid tumors (AZD7762, MK8776, prexasertib) in preventing the proliferation of cells modeling primary and MBC.

**Conclusions:**

CHK1 could be considered as an appealing novel pharmacological target for the treatment of luminal primary and MBCs.

**Supplementary Information:**

The online version contains supplementary material available at 10.1186/s13046-022-02360-y.

## Background

Breast cancer (BC) is the leading cause of cancer-related deaths in women worldwide. BC is a heterogenous disease with different molecular phenotypes. To drive the clinical implementation of BC treatment, diagnosed BCs are commonly divided into 5 different subgroups organized based on immunohistochemistry classification. These different BC types are also called clinicopathological surrogates and are commonly separated into luminal A (LumA), luminal B (LumB), epidermal growth factor receptor ERBB2/HER2-overexpressing (HER2 +), basal epithelial-like (BL), and normal-like (NL). While LumA and LumB BCs express the estrogen receptor α (ERα), the other BC subgroups contain ERα-negative tumors [1–4].

LumA and LumB represent the 70% of all BCs at the diagnosis with the ERα expression determining a favorable prognosis for patient survival, as the receptor and its signaling activated by the cognate hormone 17β-estradiol (E2) are the targets of the endocrine therapy (ET) (*i.e.,* aromatase inhibitors, ERα inhibitors as 4OH-tamoxifen and fulvestrant) [1–4]. A difference in the response of LumA and LumB tumors to ET exists being LumA tumors more sensitive than LumB tumors to 4OH-tamoxifen (Tam) (*i.e.,* the mainstay clinical treatment for ERα-positive BC). In this respect, LumB tumors show a higher expression in proliferation-related genes and a variable expression of HER2-related genes than LumA cancers. In turn, while the LumA subgroup benefits from ET alone, LumB tumors are treated with a combined approach based on ET and traditional chemotherapy [4, 5].

Resistance to ET (*i.e.,* to either aromatase inhibitors or Tam) arises in about 50% of women with luminal BC and leads to disease relapse through the formation of metastasis to secondary sites (*e.g.,* lung, bone, brain, and liver). Metastatic BC (MBC) cells become resistant to ET drugs, often still express the ERα, and are extremely difficult to manage as a standardized treatment protocol does not exist. In turn, the development of an MBC leads in most cases to patient death. Therefore, the identification of drugs that would avoid the onset of ET resistance in the primary disease as well as the identification of novel druggable pathways in the MBC setting remains two major challenges [1–5].

In this respect, our research group has followed a novel approach to identify additional drugs for primary and MBC treatment building on the assumption that drugs, which do not necessarily bind to the ERα, can induce receptor degradation, and prevent cell proliferation through alternative mechanisms [6]. After developing a screening platform to contemporarily measure different aspects of ERα signaling as well as cell proliferation [7], we demonstrated that several Food and Drug Administration (FDA)-approved drugs used for the treatment of diverse diseases can act as ‘anti-estrogen’-like compounds by inducing ERα degradation and blocking cell proliferation in cell lines modeling both the primary and the metastatic LumA phenotypes [7–13].

Because several clinical trials are in place to test the efficacy of specific inhibitors of protein kinases (*e.g.,* mTOR, AKT, IGF1-R, FGF-R, and MET) in patients with LumA and LumB (*i.e.,* ERα-positive) MBC [1], we hypothesized that kinase inhibitors could work as ‘anti-estrogen’-like compounds by contemporarily inducing ERα degradation and blocking cell proliferation.

Here, we tested a small-scale kinase inhibitor library composed of about 170 compounds in two cell lines modeling the LumA ET resistant phenotype [*i.e.,* MCF-7 with acquired (*i.e.,* Tam Res) or genetic (*i.e.,* CRISPR-Cas9 genome-edited cells to express Y537S-mutated ERα) Tam resistance] [14, 15] and identified the CHK1/CHK2 inhibitor AZD7762 as a potential ‘anti-estrogen-like’ compound.

The dissection of the AZD7762 molecular mechanism in BC cells revealed a pivotal role for the ATR:CHK1 pathway in the regulation of ERα stability and cell proliferation. These results demonstrate for the first time that CHK1 represents a novel target for the treatment of patients with LumA primary and/or metastatic ET-resistant BCs.

## Methods

### Cell culture and reagents

MCF-7, T47D-1, MDA-MB-361, BT-474 were purchased by ATCC (USA) and maintained according to the manufacturer’s instructions. 17β-estradiol (E2), DMEM (with and without phenol red), and fetal calf serum were purchased from Sigma-Aldrich (St. Louis, MO). Bradford protein assay kit as well as anti-mouse and anti-rabbit secondary antibodies were obtained from Bio-Rad (Hercules, CA). Antibodies against ERα (HC-20, rabbit), pS2 (FL-84, rabbit) were obtained from Santa Cruz Biotechnology (Santa Cruz, CA, USA); anti-phospho ERα (Ser118, mouse), anti-phospho CHK1 (Ser296 and Ser354, rabbit), and anti-phospho CHK2 (Thr68 and Ser516, rabbit), anti-CHK1 (mouse), anti-CHK2 (mouse), anti-phospho H2AX (rabbit) and anti-RPA2 (rabbit) antibodies were obtained from Cell Signaling; anti-vinculin (mouse) antibody was purchased from Sigma-Aldrich (St. Louis, MO, USA). Chemiluminescence reagent for Western blot was obtained from BioRad Laboratories (Hercules, CA, USA). Fulvestrant (*i.e.,* ICI182,780) was purchased by Tocris (USA), cycloheximide (CHX), etoposide (ETO), aphidicolin (Aph), hydroxyurea (HU), and camptothecin (CPT) were purchased from Sigma-Aldrich (St. Louis, MO, USA). The kinase library was purchased by Cayman Chemical (USA). Palbociclib, abemaciclib, prexasertib, AZD7762, and GDC-0575 were purchased by Selleck Chemicals (USA). PolarScreen™ ERα Competitor Assay Kit, Green (A15882) was acquired from Thermo Scientific. All the other products were from Sigma-Aldrich. Analytical- or reagent-grade products were used without further purification. The identities of all the used cell lines were verified by STR analysis (BMR Genomics, Italy).

### *In vitro* ERα binding assay

A fluorescence polarization (FP) assay was used to measure the binding affinity of the indicated drugs and 17β-estradiol (E2) for recombinant ERα *in vitro*. The FP assay was performed using a PolarScreen™ ERα Competitor Assay Kit, Green (A15882, Thermo Scientific) as previously reported [16].

### In-cell Western blot

In-cell Western blot was used to measure ERα levels in MCF-7, Tam Res, and Y537S cell lines. The experiments were carried on using the protocol previously described [13]. The cells were treated with kinases inhibitors in quadruplicate at a concentration of 100 nM for 48 h. Fulvestrant (*i.e.,* ICI – 100 nM) was used as the control for ERα degradation.

### In-cell Propidium Iodide (PI) staining

In-cell PI staining was used to measure DNA content in MCF-7, Tam Res, and Y537S cell lines. The experiments were carried on using the protocol previously described [13]. The cells were treated with kinases inhibitors in quadruplicate at a concentration of 100 nM for 48 h. Taxol (1 µM) was used as the control for cell proliferation.

### Measurement of ERα transcriptional activity

MCF-7 and Y537S cells were stably transfected with a plasmid containing an ERE-nanoluciferase (NLuc)-PEST reporter gene and measurement of NLuc-PEST expression (*i.e.,* ERα transcriptional activity) was performed after 24 h of compound administration as described [12, 17].

### Cell manipulation for Western blot analyses

Cells were grown in DMEM with phenol red plus 10% fetal calf serum for 24 h and then treated with the different compounds at the indicated doses for the indicated periods. Before E2 stimulation, cells were grown in DMEM without phenol red plus 10% charcoal-stripped fetal calf serum for 24 h; all kinase inhibitors were added 24 h before E2 administration. After treatment, cells were lysed in Yoss Yarden (YY) buffer (50 mM Hepes (pH 7.5), 10% glycerol, 150 mM NaCl, 1% Triton X-100, 1 mM EDTA and 1 mM EGTA) plus protease and phosphatase inhibitors. Western blot analysis was performed by loading 20–30 µg of protein on SDS-gels. Gels were run, and the proteins were transferred to nitrocellulose membranes with a Turbo-Blot semidry transfer apparatus from Bio-Rad (Hercules, CA, USA). Immunoblotting was carried out by incubating the membranes with 5% milk or bovine serum albumin (60 min), followed by incubation overnight (o.n.) with the indicated antibodies. Secondary antibody incubation was continued for an additional 60 min. Bands were detected using a Chemidoc apparatus from Bio-Rad (Hercules, CA, USA).

### Small interference RNA

MCF-7 and Y537S cells were transfected with Dharmacon Smart-Pool Oligos against either CHK1 or CHK2 and the procedure was carried out using Lipofectamine RNAi Max (Thermo Fisher) as previously reported [18].

### Cell proliferation and cell cycle assays

For growth curves, the xCELLigence DP system ACEA Biosciences, Inc. (San Diego, CA) Multi-E-Plate station was used to measure the time-dependent response to the indicated drugs by real-time cell analysis (RTCA), as previously reported [9, 12, 16]. Briefly, the number of cells (*i.e.,* normalized cell index) is directly proportional to the measured electric impedance of the cells on the well surface. Cells were seeded in E-Plates 96 in the growing medium. After overnight monitoring of growth once every 15 min, drugs were added. Cells remained in the medium until the end of the experiment. Cellular responses were then recorded once every 15 min for a total time of 5 days. For cellular co-treatment with the indicated drugs and Tam, cells were plated (2000 cell/well) in triplicate in 96-well plates and treated with different concentrations of both drugs. Seven days after initial compound administration treatments were refreshed and after 12 days cells were stained with Crystal Violet and solubilized with SDS 1%. Absorbance was then read in the Tecan Spark microplate reader at 595 nM. Next, the synergy index was calculated with Combenefit freeware software [19].

For cell cycle analysis, Nicoletti’s protocol was followed [20]. Briefly, the cell pellet was resuspended in 500 µl of DNA staining buffer (0.19 M Na_2_HPO_4_, 0.004% Triton X-100, pH 7.8 and 20 µg/ml of propidium iodide). Cells were incubated for 30 min at room temperature in the dark. Finally, 20.000 total events on a linear scale were acquired and the percentage of each cell cycle phase was calculated by a proper electronic marker. Samples were acquired with a CytoFlex Flow Cytometer (Beckman Coulter) equipped with 488 nm laser source. Cell cycle analysis was performed using CytExpert v.2.4 software (Beckman Coulter). Doublet discrimination was performed by an electronic gate on FL2-Area vs. FL2-Height parameters.

### Bromodeoxyuridine incorporation assay

Bromodeoxyuridine (BrdU) was added to the medium in the last 30 min of growth, and the cells were then fixed and permeabilized. Histones were dissociated with 2 M HCl as previously described [21]. BrdU-positive cells were detected with anti-BrdU primary antibody diluted 1:100 (DAKO; Santa Clara, CA, USA) and Alexa488-conjugated anti-mouse antibody diluted 1:100 (Thermo Fisher Scientific; Waltham, MA, USA). Both antibodies were incubated with the cells for 1 h at room temperature in the dark. BrdU fluorescence was measured using a CytoFlex flow cytometer, and S-phase analysis was performed with CytExpert v 2.3 software (Beckman Coulter, Brea, CA, USA). All samples were counterstained with propidium iodide (PI) for DNA/BrdU bi-parametric analysis.

### 3D cell cultures

Tumor spheroid formation was performed as previously reported [7, 9]. Briefly, MCF-7 and Y537S cells were seeded (10,000 cells/well) in ultra-low attachment surface 24-well-plates (Sigma-Aldrich) with 1 ml/well in growing condition for 24 h. Next, using an optical microscope, pictures had been taken for each well in untreated conditions (*i.e.,* time 0). At time 0, cells were treated in quadruplicate with the indicated compounds and with vehicle (DMSO). After 48 h, the cell culture medium was changed using a 70 µm nylon sterile cell strainer for each condition to maintain spheroids with a diameter greater than 70 µm and to remove dead cells and spheroids with a diameter smaller than 70 µm. Contemporarily, the treatment was repeated. Seven days post initial drug administration, at least 3 pictures for each well had been taken. The number of spheroids has been quantitated using the freeware software Image J by measuring the surface area occupied by each spheroid in each picture taken for each condition. Spheroids, which were reduced to debris because of the treatment, were excluded from the analysis.

Alginate-based cultures of MCF-7 and Y537S cells were prepared according to [22, 23]. To generate the alginate spheres, alginate powder (ADD121—BAIOCCO S.R.L., Concorezzo, Italy) was dissolved in NaCl 0.9% w/v solution at a final concentration of 1% w/v (Alginate Solution) for 24 h at room temperature and then filtered through a 0.22 µm filter. In parallel, cells were resuspended in a complete growing medium (Cell suspension). After that, alginate solution and cell suspension were mixed in a 1:1 ratio to obtain 2000 cells/15 µl in alginate solution *plus* cell suspension (*i.e.,* cell:alginate suspension). After vortexing, the cell:alginate suspension has been transferred into a 5 ml syringe with a 21 gauge needle and alginate spheres containing cells have been generated by pouring drop to drop in a 0.5 M CaCl_2_ solution the cell:alginate suspension present in the syringe. After 5 min, alginate spheres containing cells were first washed with growing medium plus 5 mM CaCl_2_ and transferred into tissue culture dishes by using sterile tweezers. Notably, alginate cultures have been maintained in the growing medium with the addition of 5 mM CaCl_2_ solution. For growth curve analyses, 24 h after plating in 6 well plates (10 spheres per plate), alginate-based cultures were treated in triplicate with the different compounds for a total time of 7 days. Measurement of cell number was performed by harvesting the alginate-based cultures and then by dissolving the spheres in citric acid (100 mM in NaCl 0.9% w/v) through vortexing the solution. After centrifugation at maximum speed for 2 min, cells were lysed in YY buffer and total proteins have been quantitated with Bradford assay (please, see above).

### Statistical analysis

Statistical analysis was performed using the InStat version 8 software system (GraphPad Software Inc., San Diego, CA). Densitometric analyses were performed using the freeware software Image J by quantifying the band intensity of the protein of interest with respect to the relative loading control band (*i.e.,* vinculin) intensity. The *p* values and the used statistical test are given in figure captions.

## Results

### Kinase inhibitor screen identifies CHK1/CHK2 as potential novel targets for ERα-positive BCs

**Fig. 1:**
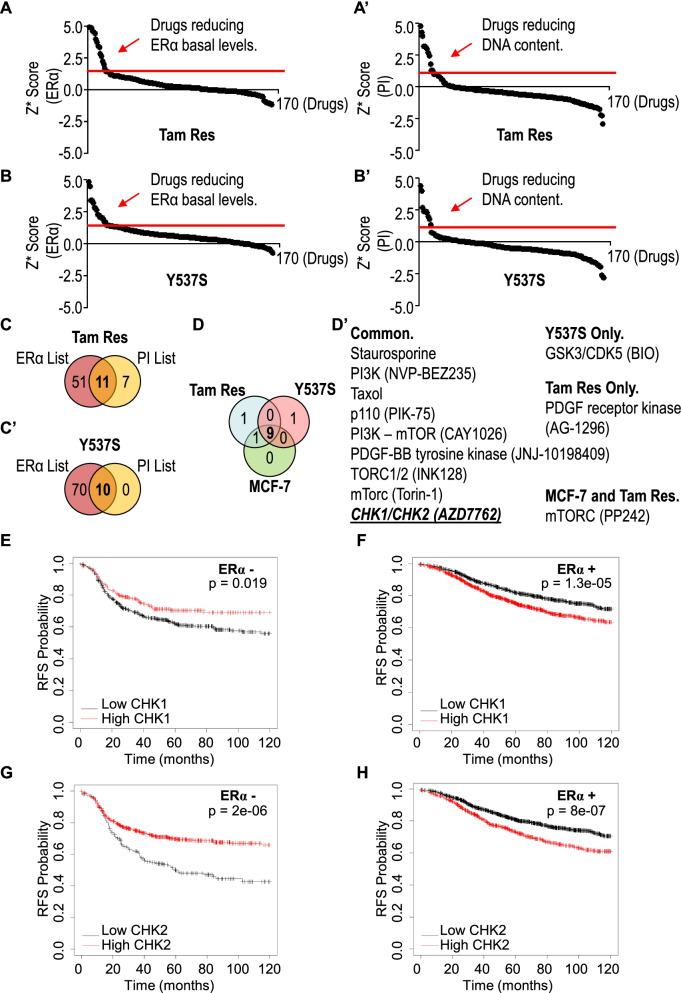
Identification of kinase inhibitor influencing ERα levels and cell proliferation in luminal primary and metastatic breast cancer cells. Robust *Z* scores (Z*) graphs for kinase inhibitor-treated samples (100 nM final concentration) for 48 h in Tam Res and Y537S cells. The ERα levels were detected by in-cell WB (A and B) while cell number was detected by in-cell propidium iodide (PI) staining (**A’** and **B’**); red arrows indicate the Z* for inhibitors considered as positive hits. Red lines indicate the threshold used for analysis. Venn diagrams showing the positive hits for ERα and PI lists in Tam Res (**C**) and Y537S (**C’**) cells. (**D**) Venn diagram showing the combined positive hits of the inhibitors (explicated in **D’** – compound names are given in brackets) in the indicated cell lines. Kaplan–Meier plots show the relapse-free survival (RFS) probability in women carrying ERα-negative (**E** and **G**) or ERα-positive (**F** and **H**) as a function of CHK1 (**E** and **F**) or CHK2 (**G** and **H**) mRNA levels. All possible cutoff values between the lower and upper quartiles are automatically computed (*i.e.,* auto select best cutoff on the website), and the best performing threshold is used as a cutoff [27]. Details of the parameters of the curves are given in supplementary table 3. Significant differences between the RFS are given as *p-*value in each panel

To evaluate the impact of kinases in the regulation of ERα stability and cell proliferation, a small-scale library of kinase inhibitors was tested in ductal carcinoma cells (*i.e.,* MCF-7 cells) modeling primary BC and MBC. Initially, we measured the ability of each kinase inhibitor to reduce both ERα intracellular content and cell proliferation by using *in-cell* Western blot (WB) and propidium iodide (PI) staining [10, 13] both in MFC-7 cells adapted to grow in the presence of Tam (Tam Res) [14] and in MCF-7 cells genetically engineered to express the Y537S ERα mutant (Y537S), which is a hyperactive receptor variant conferring resistance to ET (*i.e.,* aromatase inhibitors and Tam) [15, 24]. We calculated the robust *Z* score (Z*) [25, 26] and set thresholds (red lines in Fig. 1A and A’) for the definition of positive hits (Fig. 1A, A’, B and B’) to identify groups of kinase inhibitors reducing either ERα intracellular levels or basal cell proliferation in both cell lines. By using these limits, we shortlisted 11 and 10 drugs that contemporarily affected both ERα intracellular levels and cell proliferation in Tam Res and Y537S cells, respectively (Fig. 1C and C’). Next, we repeated the same experiments to test the ability of these drugs to impact both ERα intracellular levels and cell proliferation in parental MCF-7 cells, which are the most widely used cells modeling primary BC. As shown in Fig. 1D, the Venn diagram indicates that 9 kinase inhibitors (Fig. 1D’ in brackets) were implicated in reducing both ERα levels and cell proliferation in all the 3 tested cell lines while only 3 kinase inhibitors specifically affected either 1 or 2 cell lines (Fig. 1D and D’ in brackets).

Inspection of the identified kinase inhibitors revealed that most of the common drugs affecting the measured parameters targeted the PI3K/mTOR/AKT pathway, thus supporting the notion that this pathway is a drug target for the treatment of MBC [1]. Interestingly, we also found the CHK1/CHK2 inhibitor (*i.e.,* AZD7762) in this list (Fig. 1D’).

Prompted by these results, we next evaluated whether different levels of CHK1 and CHK2 mRNA expression could impact the survival of women carrying ERα-negative or ERα-positive BCs. Kaplan–Meier curves were retrieved by the Kaplan–Meier Plotter database (https://kmplot.com/analysis/) [27] and showed that women with ERα-negative BC (Fig. 1E, G and Supplementary Table 3) display an increased relapse-free survival (RFS) rate when the tumor expresses high levels of either CHK1 or CHK2 while, on the contrary, women with ERα-positive BCs expressing low levels of either CHK1 or CHK2 have a high RFS probability with respect to those patients expressing high CHK1 and CHK2 mRNA levels (Fig. 1F, H and Supplementary Table 3).

### CHK1 and ERα activation states are correlated in ERα-positive BC cell lines

**Fig. 2:**
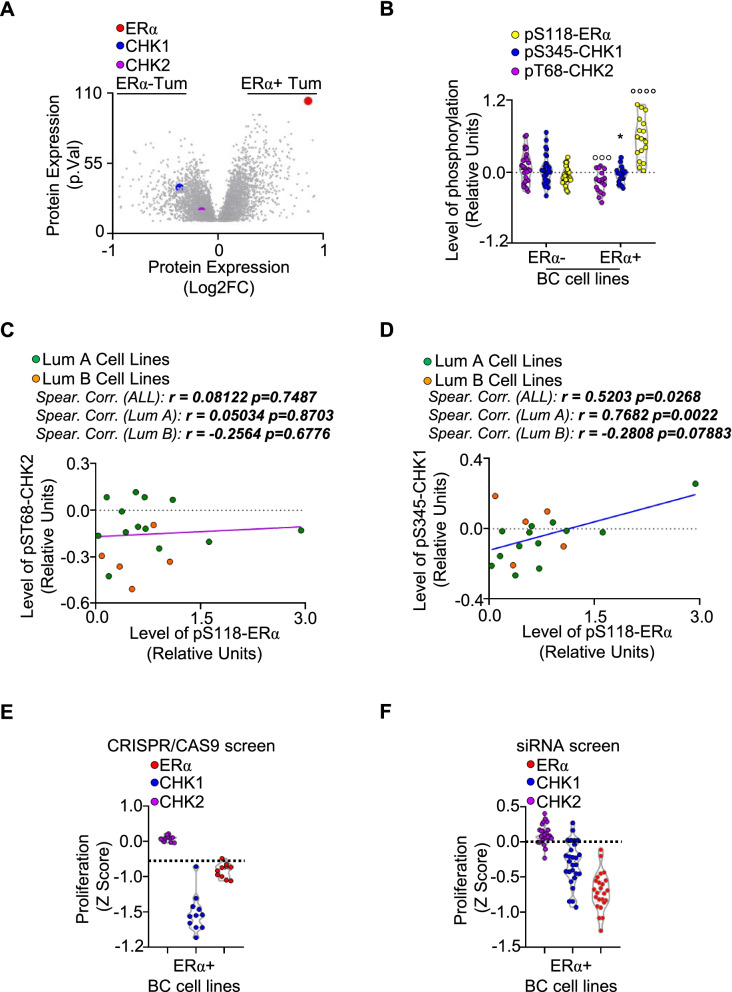
Correlation between ERα and CHK1 and CHK2 activation status. **A** Volcano plot showing the protein expression as a function of the *p*-value of the indicated proteins (*i.e.,* ERα—red, CHK1—blue and CHK2 – purple) in ERα-negative or ERα-positive tumors as indicated in the breast cancer landscape [3] and downloaded by https://www.breastcancerlandscape.org/. **B** Level of ERα S118 phosphorylation (yellow), CHK1 S345 phosphorylation (blue), and CHK2 T68 phosphorylation (purple) in ERα-negative or ERα-positive breast cancer cell lines. Significant differences were obtained by unpaired two-tailed Student’s t-test. °°°° (*p* < 0.0001) indicates significant differences in ERα S118 phosphorylation between ERα-negative or ERα-positive breast cancer cell lines. °°° (*p* < 0.001) indicates significant differences in CHK2 T68 phosphorylation between ERα-negative or ERα-positive breast cancer cell lines. * (*p* < 0.05) indicates significant differences in CHK1 S345 phosphorylation and CHK2 T68 phosphorylation in ERα-positive breast cancer cell lines. Linear regression and Spearman Correlation values (r) between ERα S118 phosphorylation and CHK2 T68 phosphorylation (**C**) or ERα S118 phosphorylation and CHK1 S345 phosphorylation (**D**) in ERα-positive breast cancer cell lines classified as luminal A (LumA – green) or luminal B (LumB – orange). r and *p* values are given in the main panel. *Z* scores for the antiproliferative effects of either ERα (red), CHK1 (blue), or CHK2 (purple) depletion by CRISPR/CAS9 (**E**) or small interference RNA (siRNA) (**F**) in ERα-positive breast cancer cell lines. The dotted lines represent the threshold for cell line sensitivity in each kind of procedure. Plots in (**B**-**F**) have been generated by downloading the experimental data by the Broad Institute through the DepMap portal https://depmap.org/portal. Each dot of the plots in (**B**-**F**) represents the value of the indicated parameter in a single breast cancer cell line. Crude data are given in supplementary table 1

Next, we inspected CHK1 and CHK2 expression levels in specific datasets for ERα-negative and ERα-positive tumors provided by the BC quantitative proteome and proteogenomic landscape (https://www.breastcancerlandscape.org/). These datasets contain the integrated characterization and classification of 45 different breast tumors in terms of proteomics, transcriptomics, metabolomics, and phosphoproteomics [3]. Unexpectedly, both CHK1 and CHK2 protein expression levels are significantly higher in ERα-negative tumors than in ERα-positive ones (Fig. 2A).

Therefore, we hypothesized that a differential activation status of CHK1 and CHK2 could be present in ERα-positive BCs compared with ERα-negative tumors. To assess this hypothesis, we extrapolated from the DepMap portal (https://depmap.org/portal) the data regarding the phosphorylation status of i) CHK1 at Ser345 (S345), which is ATR-dependent and determines CHK1 activation [28], ii) CHK2 at Thr68 (T68), which is ATM-dependent and determines CHK2 activation [29], and iii) ERα on Ser118 (S118), which directly measures the receptor activation [30]. These analyses were performed in all the profiled BC cell lines stratified based on ERα expression. As expected [31], the phosphorylation of ERα at S118 is significantly higher in ERα-positive BC cell lines with respect to the ERα-negative BC cell lines. On the contrary, while no differences have been detected among ERα-negative and ERα-positive BC cell lines in the phosphorylation of CHK1 at S345, the phosphorylation of CHK2 at T68 was significantly reduced in ERα-positive BC cell lines with respect to the ERα-negative ones (Fig. 2B).

Interestingly, data also showed that the phosphorylation of CHK1 at S345 is significantly higher than the phosphorylation of CHK2 at T68 residue in ERα-positive BC cell lines (Fig. 2B). No significant differences were found in the phosphorylation status of the two kinases in ERα-negative cell lines (Fig. 2B). Overall, these data indicate that CHK1 activation is basally higher than CHK2 activation in ERα expressing BC cells.

Further analysis of the activation status of either CHK1, CHK2 or ERα in ERα-positive BC cells revealed that no correlation between CHK2 and ERα activation exists in ERα-positive cells even if the cell lines are stratified as belonging to the LumA or LumB tumor phenotype (Fig. 2C). On the contrary, we observed a significant positive correlation (*r* = 0.5203 *p* = 0.0268) between the phosphorylation of CHK1 at S345 and the phosphorylation of ERα at S118 (Fig. 2D). Notably, this correlation was even more evident (*r* = 0.7682 *p* = 0.0022) in LumA cell lines and was not observed in LumB cell lines (Fig. 2D). Thus, these data suggest that CHK1 activation is correlated with ERα activation in LumA tumors.

Prompted by these results, we next studied if either CHK1 or CHK2 depletion was lethal for ERα-positive BC cells by interrogating the CRISPR/CAS9 and siRNA screen datasets available in the DepMap portal. These datasets consider a gene to be essential for survival (*i.e.,* its depletion prevents cell proliferation) if the score is ≤ -0.5 for the CRISPR/CAS9 screen and ≤ 0 for the siRNA screen [32]. As a control, we extracted the data regarding the effect of ERα gene silencing on cell proliferation. As expected, depletion of the ERα is lethal for ERα-positive BC cells both in the CRISPR/CAS9 (Fig. 2E) and in the siRNA (Fig. 2F) screen. While CHK2 depletion did not change the proliferation of ERα-positive BC cell lines in both high-throughput screens, CHK1 depletion induced by CRISPR/CAS9- or siRNA-mediated experiments reduced the cell viability of ERα-positive BC cell lines (Fig. 2E and F).

Overall, these data indicate that i) CHK1/CHK2 inhibition could be a valuable strategy to prevent ERα-positive BC progression, ii) CHK1 and ERα activations are correlated in ERα-positive BC cell lines, and iii) CHK1 downmodulation blocks the proliferation of ERα-positive BC cells.

### The ATR and CHK1 inhibition controls ERα stability and cell proliferation in BC cells

These *in silico* observations together with the results of the screening experiments strongly suggest a molecular link among CHK1, CHK2, and ERα functions in BC cells. Therefore, we next validated the ability of AZD7762 to reduce receptor intracellular content and cell proliferation.

**Fig. 3:**
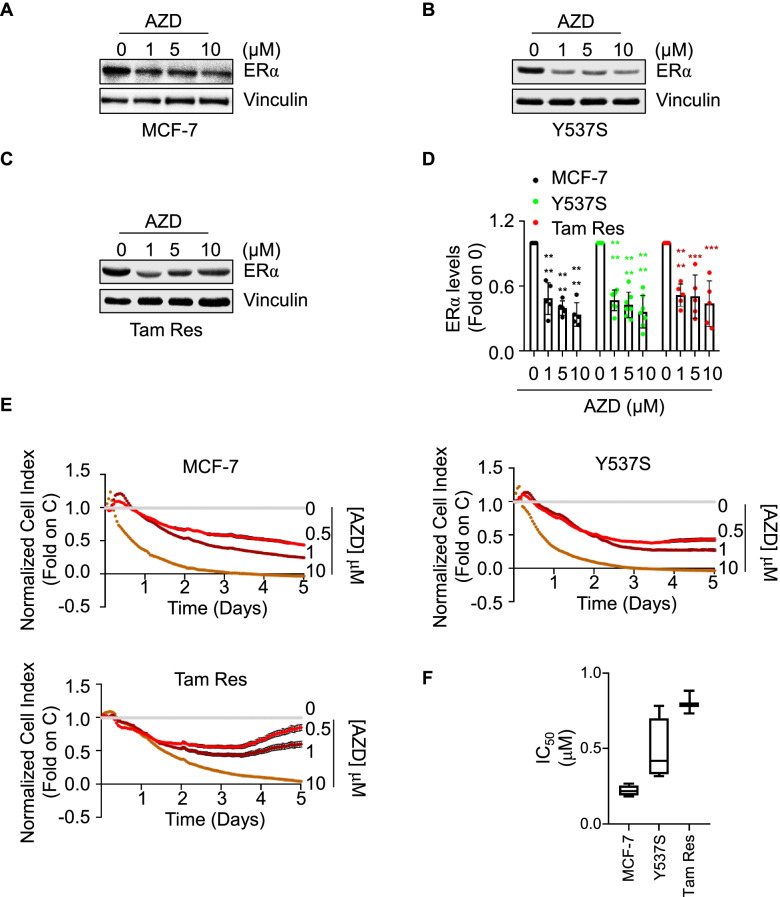
Validation of the AZD7762 effect in MCF-7, Y537S, and Tam Res cells. Western blot (**A**-**C**) and relative densitometric (**D**) analyses of ERα expression levels in (**A**) MCF-7, (**B**) Y537S, and (**C**) Tam Res cells treated for 24 h with the indicated doses of AZD7762 (AZD). The loading control was done by evaluating vinculin expression in the same filter. Significant differences with respect to control (0) were obtained by unpaired two-tailed Student’s t-test. Data show the mean ± the standard deviations. **** *p* < 0.0001; *** *p* < 0.001. The number of replicates is given as solid dots in the bar graph. **E** Growth curve analyses in MCF-7, Y537S, and Tam Res cells were performed as indicated in the material and method section for 5 days with the indicated doses of AZD7762 (AZD). The graphs show the normalized cell index (*i.e.,* cell number), which is detected with the xCelligence DP device and calculated at each time point with respect to the control sample. Each sample was measured in a quadruplicate. For details, please see the material and methods section. **F** The inhibitor concentration 50 (IC_50_) was calculated for each cell line at 5 days after initial treatment

Initial experiments were performed to verify the impact of CHK1/CHK2 inhibition on the control of ERα intracellular levels both in parental MCF-7, Tam Res, and Y537S cells. As expected, AZD7762 (AZD) treatment prevented the DNA damaging agent etoposide (ETO)-dependent induction of CHK1 and CHK2 activation in the tested cell lines (Supplementary Fig. 1A and B). Twenty-four hours of AZD administration to BC cells significantly reduced ERα intracellular content in a dose-dependent manner in each tested cell line (Fig. 3A-D). In addition, real-time growth curve analyses showed that AZD exerted an anti-proliferative effect as a function of the tested dose in both MCF-7, Y537S, and Tam Res (Fig. 3E) cells. Quantitation of the inhibitory concentration 50 (IC50) at 5 days after AZD administration indicated that all the IC_50_ values are in the low μM range and showed that the different cell lines have a different sensitivity to the drug (*i.e.,* MCF-7 > Y537S > Tam Res) with cells resistant to Tam being the less sensitive to AZD possibly because Tam resistance in BC cells is accompanied by an increased expression of multidrug resistance-associated proteins [33].

**Fig. 4:**
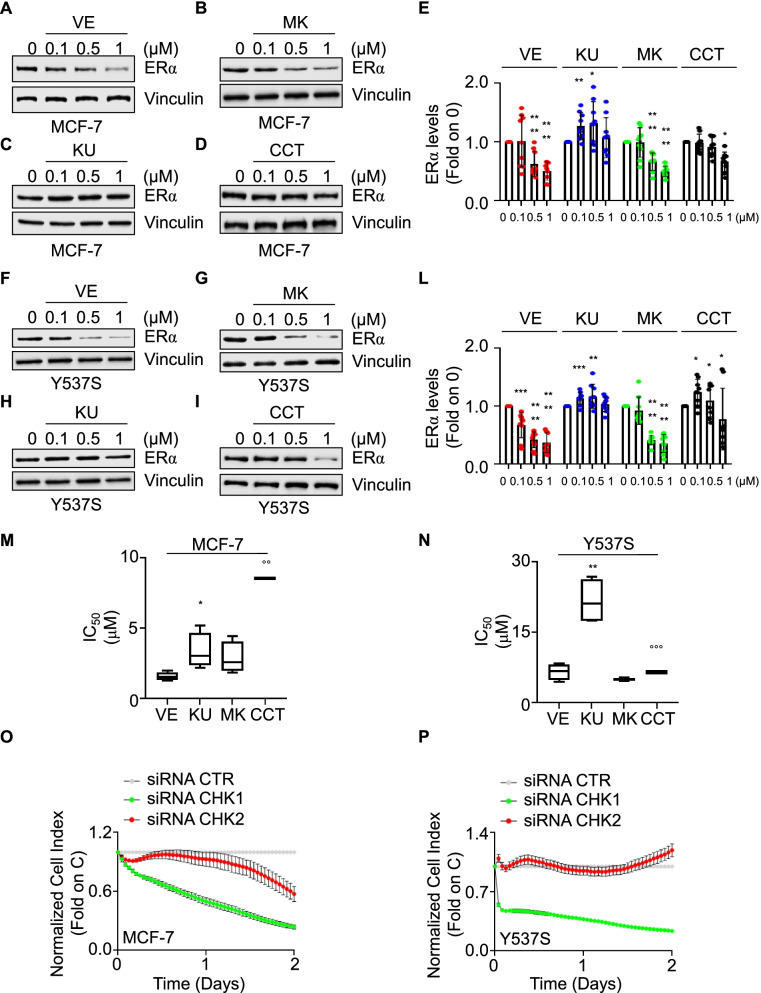
Dissection of the pathway inducing ERα degradation and preventing proliferation in MCF-7 and Y537S cells.Western blot and relative densitometric (**E** and **L**) analyses of ERα expression levels in (**A**-**D**) MCF-7 and (**F**–**H**) Y537S cells treated for 24 h with the indicated doses of the specific inhibitors of either CHK1 (*i.e.,* MK8776—MK), CHK2 (*i.e.,* CCT241533—CCT), ATR (*i.e.,* VE822—VE) or ATM (*i.e.,* KU60019—KU). The loading control was done by evaluating vinculin expression in the same filter. Significant differences with respect to control (0) were obtained by unpaired two-tailed Student’s t-test. Data show the mean ± the standard deviations. **** *p* < 0.0001; *** *p* < 0.001; ** *p* < 0.01; * *p* < 0.05. The number of replicates is given as solid dots in the bar graph. (**M**–**N**) The inhibitor concentration 50 (IC_50_) was calculated for the indicated cell lines at 5 days after initial treatment with the indicated inhibitors. Significant differences were obtained by unpaired two-tailed Student’s t-test. Data show the mean ± the standard deviations. * (*p* < 0.05) and ** (*p* < 0.01) indicate significant differences between VE and KU IC_50_ while °° *p* < 0.01 and °°° p < 0.001 indicate significant differences between MK and CCT IC_50_. Growth curve analyses in MCF-7 (**O**) and Y537S (**P**) cells were performed as indicated in the material and method section for 2 days after the cell transfection with the small interference RNA (siRNA) oligonucleotides against either CHK1 or CHK2. The graphs show the normalized cell index (*i.e.,* cell number), which is detected with the xCelligence DP device and calculated at each time point with respect to the control sample. Each sample was measured in a quadruplicate. For details, please see the material and methods section. Controls for CHK1 and CHK2 siRNA experiments are available in supplementary Fig. 1C and D

Because AZD inhibits both CHK1 and CHK2, we next sought to dissect the contribution of each kinase in the regulation of receptor stability. Dose–response curves were performed in both parental and Y537S cells treated not only with both the specific inhibitors of CHK1 (*i.e.,* MK8776 -MK) and CHK2 (*i.e.,* CCT241533—CCT) but also with the specific inhibitors of both ATR (*i.e.,* VE822—VE) and ATM (*i.e.,* KU60019—KU), which are the upstream kinases regulating CHK1 and CHK2 activation [34], respectively. As expected, all the tested inhibitors worked in both cell lines (Supplementary Fig. 2). Results indicate that the 24 h treatment of MCF-7 (Fig. 4A-E) and Y537S (Fig. 4F-L) cells with either VE or MK induced a dose-dependent reduction in ERα intracellular content (Fig. 4A, B, F, G, E and L; see also Fig. 7) while administration of KU and CCT determined an inconsistent dose-dependent modification in receptor levels (Fig. 4C, D, E, H, I and L; see also Fig. 7).

Growth curves analyses were performed in both parental MCF-7 and Y537S cells to test the anti-proliferative effects of ATR, ATM, CHK1, and CHK2 inhibitors. The calculation of the IC_50_ values at 5 days for each inhibitor revealed that the inhibition of ATM by KU determined a lower antiproliferative effect than the one achieved by ATR inhibition by VE. In addition, the IC_50_ for the CHK1 inhibitor MK was lower than that of the CHK2 inhibitor CCT in both MCF-7 and Y537S cells (Fig. 4M and N). Accordingly, in both MCF-7 and Y537S cells the siRNA-mediated depletion of CHK1 caused a time-dependent impairment in cell proliferation while siRNA-mediated depletion of CHK2 did not affect the cell basal growth rate (Fig. 4O, P and Supplementary Fig. 1C and D).

**Fig. 5:**
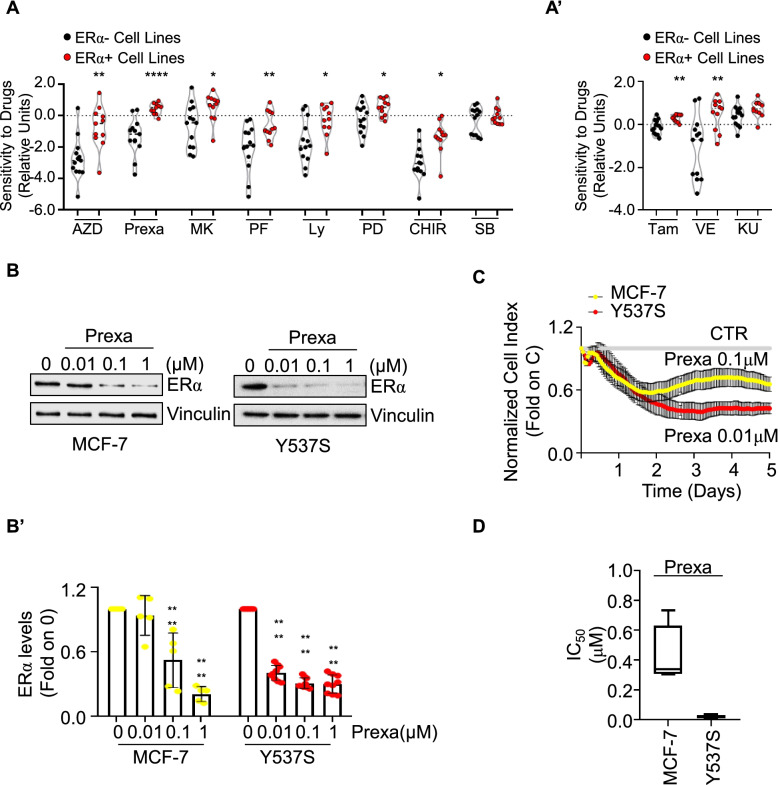
Impact of clinically relevant CHK1 inhibitors in inducing ERα degradation and preventing proliferation in MCF-7 and Y537S cells. **A** Sensitivity scores of ERα-negative (black) and ERα-positive (red) breast cancer cell lines to AZD7762 (AZD), prexasertib (Prexa), MK8776 (MK), PF-477736 (PF), Ly-2603618 (Ly), PD-407824 (PD), CHIR-124 (CHIR), and SB-218078 (SB). **A’** Sensitivity scores of ERα-negative (black) and ERα-positive (red) breast cancer cell lines to 4OH-Tamoxifen (Tam), VE822 (VE), and KU60019 (KU). * (*p* < 0.05), ** (*p* < 0.01) and **** (*p* < 0.001) indicate significant differences to drugs among ERα-negative or ERα-positive breast cancer cell lines. The graphs have been generated by downloading the experimental data from the Broad Institute through the DepMap portal https://depmap.org/portal. Each dot of the plot in (**A** and **A’**) represents the value of the indicated parameter in a single breast cancer cell line. Crude data are given in supplementary table 1. Western blot (**B**) and relative densitometric analyses (**B’**) of ERα expression levels in MCF-7 (yellow) and Y537S (red) cells treated for 24 h with the indicated doses of prexasertib (Prexa). The loading control was done by evaluating vinculin expression in the same filter. Significant differences with respect to control (0) were obtained by unpaired two-tailed Student’s t-test. Data show the mean ± the standard deviations, **** *p* < 0.0001. The number of replicates is given as solid dots in the bar graph. **C** Growth curve analyses in MCF-7 (yellow) and Y537S (red) cells were performed as indicated in the material and method section for 5 days with different doses of prexasertib (Prexa). The graph shows only one concentration for each cell line and the normalized cell index (*i.e.,* cell number), which is detected with the xCelligence DP device and calculated at each time point with respect to the control sample. Each sample was measured in a quadruplicate. For details, please see the material and methods section. **D** The inhibitor concentration 50 (IC_50_) was calculated for each cell line at 5 days after initial treatment

Next, to evaluate if CHK1 inhibition could be a possible pharmacological target [35–37] also in ERα-positive BCs, we inspected the DepMap datasets regarding the sensitivity of diverse BC cell lines to diverse CHK1 inhibitors that are in clinical trials (*i.e.,* AZD; MK; prexasertib – Prexa; PF-477736 – PF; Ly-2603618 – Ly; PD-407824 – PD; CHIR-124 – CHIR; SB-218078—SB) [35–37]. The obtained results were stratified according to ERα expression. As controls, we also included the sensitivity of the different cell lines both to Tam, to the ATR inhibitor VE and to the ATM inhibitor KU (to date, no CHK2 inhibitor has been tested in the DepMap database). Data revealed that ERα-positive BC cell lines are significantly more sensitive to all the CHK1 inhibitors analyzed (except for SB) than ERα-negative BC cell lines (Fig. 5A). The sensitivity to ATR inhibition is higher in ERα-positive than in the ERα-negative BC cell lines while no differences have been observed for BC cell lines treated with the ATM inhibitor KU. ERα-positive BC cell lines show increased sensitivity to Tam with respect to the ERα-negative BC cell lines (Fig. 5A’).

Prompted by these results, we next studied the impact of two additional CHK1 inhibitors [*i.e.,* Prexa and GDC-0575 (GDC)] [35–37] on the control of ERα stability and cell proliferation in parental and Y537S cells. As shown in Fig. 5B, Prexa administration to both MCF-7 and Y537S cells dose-dependently decreased ERα intracellular content. Accordingly, Prexa reduced the cell proliferation of both BC cell lines with an IC_50_ value in the low μM range with Prexa being more effective in Y537S than in MCF-7 cells (Fig. 5C and D). Notably, similar results were obtained by treating MCF-7 and Y537S cells with GDC (Supplementary Fig. 3).

Overall, these data demonstrate that the integrity of the ATR:CHK1 axis is required to maintain ERα intracellular content and to fuel cell proliferation in LumA BC cells modeling primary and MBC.

### The ATR:CHK1-dependent control of ERα intracellular concentration

**Fig. 6:**
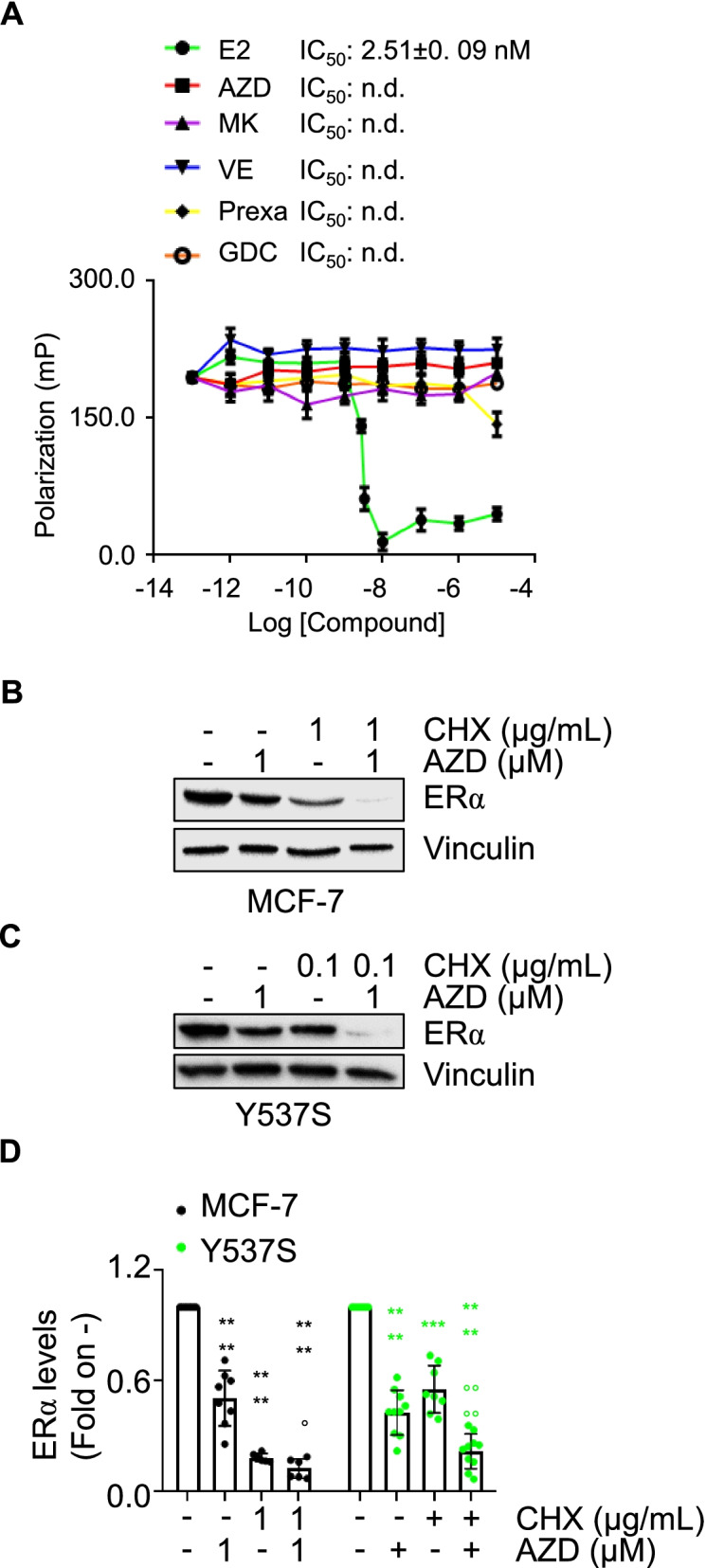
Evaluation of the inhibitor-dependent mechanism for ERα degradation. **A** *In vitro* ERα competitive binding assays for MK8776 (MK), CCT241533 (CCT), VE822 (VE), KU60019 (KU), AZD7762 (AZD) prexasertib (Prexa), GDC-0575 (GDC), and 17β-estradiol (E2) were performed at different doses of the compounds and using a florescent E2 as the tracer. Relative inhibitor concentration 50 (IC_50_, *i.e.,* K_d_) is given in the graph. The experiment was performed twice in quintuplicate. Western blot (**B**-**C**) and relative densitometric analysis (**D**) of ERα levels in MCF-7 (**B**) and Y537S (**C**) cells pre-treated with cycloheximide (CHX) at the indicated doses for 6 h and then treated with AZD7762 at the indicated dose for 24 h. The loading control was done by evaluating vinculin expression in the same filter. The number of replicates is given as solid dots in the bar graph. Significant differences with respect to CTR sample are calculated by Student t-test and indicated by **** (*p*-value < 0.0001). Significant differences with respect to the CHX sample are calculated by Student t-test and indicated by ° (*p*-value < 0.05), and °°°° (*p*-value < 0.0001)

Ligand-induced ERα reduction in BC cells can be due to the ability of the ligand to directly bind to the receptor [38]. In turn, ERα binding assays were performed with different doses of AZD, MK, VE, Prexa, GDC, and E2, to test whether these kinase inhibitors could directly bind ERα *in vitro*. Only E2 (Fig. 6A) was able to displace the fluorescent E2, used as a tracer for the recombinant purified ERα, with an IC_50_ (*i.e.,* K_d_) value of approximately 2.5 nM, as previously reported [9]. Next, we tested if kinase inhibition could impact on ERα protein turnover rate. MCF-7 and Y537S cells were pre-treated with the protein synthesis inhibitor cycloheximide (CHX) for 6 h before 24 h of AZD administration. As expected, AZD and CHX reduce ERα levels. Notably, because CHX-induced reduction in ERα levels occurred at lower concentrations in Y537S cells (*i.e.,* 0.1 µg/mL) than in MCF-7 cells (*i.e.,* 1 µg/mL), we used these doses to better distinguish the ability of AZD to reduce receptor levels also under CHX administration. Interestingly, in both cell lines, AZD was able to further influence the CHX-dependent reduction in ERα intracellular content (Fig. 6B-D). These results indicate that kinase inhibitors do not bind ERα *in vitro* and, possibly, control ERα abundance at the post-translational level.

**Fig. 7:**
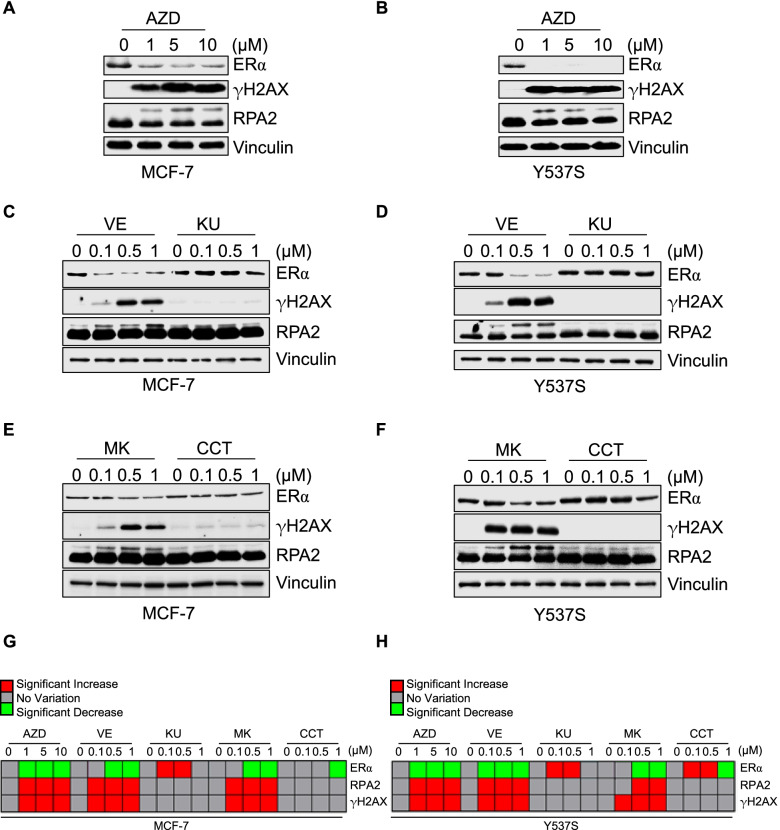
Inhibitor-dependent effect on DNA damage and replication stress in MCF-7 and Y537S cells.Western blot and relative densitometric (**G** and **H**) analyses of ERα, RPA2 and phosphorylated γH2AX expression levels in MCF-7 (**A**, **C**, and **E**) and Y537S (**B**, **D**, and **F**) cells treated for 24 h with the indicated doses of the specific inhibitors of either CHK1 (*i.e.,* MK8776—MK), CHK2 (*i.e.,* CCT241533—CCT), ATR (*i.e.,* VE822—VE) or ATM (*i.e.,* KU60019—KU) as well as with AZD7762 (AZD). The loading control was done by evaluating vinculin expression in the same filter. Significant differences are given in the heatmaps (**G** and **H**) with red being a significant increase and green being a significant decrease with respect to control (0). Analyses were performed by using the unpaired two-tailed Student’s t-test. Data are the mean ± the standard deviations, and blots show representative images of three different experiments. Histograms relative to the heatmaps are available in supplementary Fig. 7

ATR and CHK1 are involved in the replication stress response, their inhibition induces high levels of replication stress and, consequently, DNA damage [39]. Results reported here have shown that the inhibition of the ATR:CHK1 axis decreases ERα intracellular content (Fig. 4) at the post-translational level (Fig. 6). Therefore, we hypothesized a connection between replication stress and/or DNA damage and the CHK1-dependent regulation of ERα stability. Dose-dependent experiments were repeated in MCF-7 and Y537S cells treated for 24 h with different doses of AZD, VE, KU, MK, and CCT, and the receptor levels were evaluated in parallel with the detection of both H2AX and RPA2 phosphorylation [*i.e.,* a well-known DNA damage marker and a replication stress marker, respectively [39]]. Results indicate that AZD, VE, and MK inhibitors induced both ERα degradation and H2AX phosphorylation [*i.e.,* the H2AX phosphorylation at S19 being named γH2AX, hereafter] and RPA2 phosphorylation [*i.e.,* the shift in RPA2 molecular weight [40]]. On the contrary, KU and CCT did not significantly affect the phosphorylation of either H2AX or RPA2 as well as ERα intracellular content (Fig. 7A-H). Overall, these data suggest that the ATR:CHK1-dependent ERα degradation occurs in parallel with the induction of both DNA damage and replication stress.

**Fig. 8:**
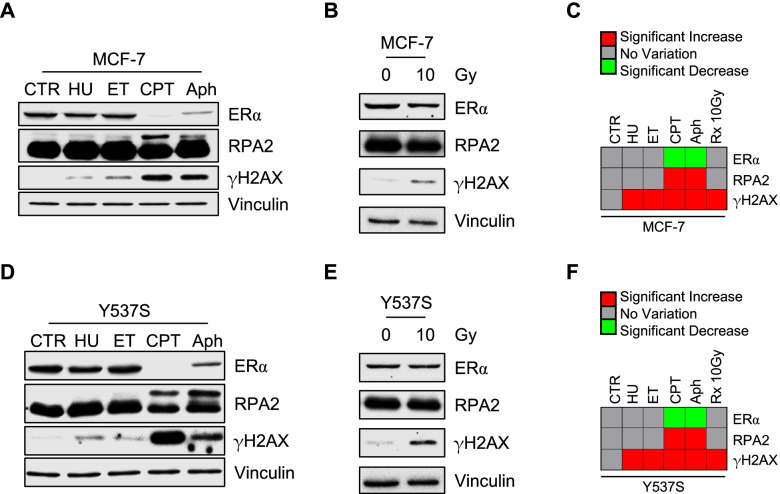
Replication stress and X-rays dependent effect on ERα levels in MCF-7 and Y537S cells. Western blot and relative densitometric (**C** and **F**) analyses of ERα, RPA2 and γH2AX expression levels in MCF-7 (**A**-**C**) and Y537S (**B**-**F**) cells treated for 24 h with hydroxyurea (HU—200 mM), etoposide (ETO – 1 μM), camptothecin (CPT – 100 nM) or aphidicolin (Aph – 5 μM). For X-rays, cells were lysed 4 h after irradiation and analyzed by Western blotting. The loading control was done by evaluating vinculin expression in the same filter. Significant differences are given in the heatmaps (**C** and **F**) with red being a significant increase and green being a significant decrease with respect to control (CTR). Analyses were performed by using the unpaired two-tailed Student’s t-test. Data are the mean ± the standard deviations, and blots show representative images of three different experiments. Histograms relative to the heatmaps are available in supplementary Fig. 7

To understand if receptor degradation occurs as a function of DNA damage and/or replication stress, MCF-7 and Y537S cells were treated with several inducers of DNA damage and replication stress (*i.e.,* hydroxyurea—HU, ETO, camptothecin—CPT, and aphidicolin—Aph) [41]. While all the used compounds were able to increase γH2AX although to a different extent, only CPT and Aph increased RPA2 phosphorylation levels and significantly reduced ERα intracellular content (Fig. 8A, C, D, and F). On the contrary, the DNA damage-inducing drugs HU and ET did not modify either RPA2 phosphorylation levels or ERα intracellular content.

To further test the effects of a well-known DNA damage-inducing agent [39, 41], MCF-7 and Y537S cells were irradiated with 10 Gy of X-rays. Results show that irradiation did not affect ERα expression levels in any of the tested cell lines (Fig. 8B, C, E, and F). Overall, present results suggest that ERα levels decrease only when replication stress is induced.

### The impact of the ATR:CHK1 pathway inhibition on E2:ERα signaling

**Fig. 9:**
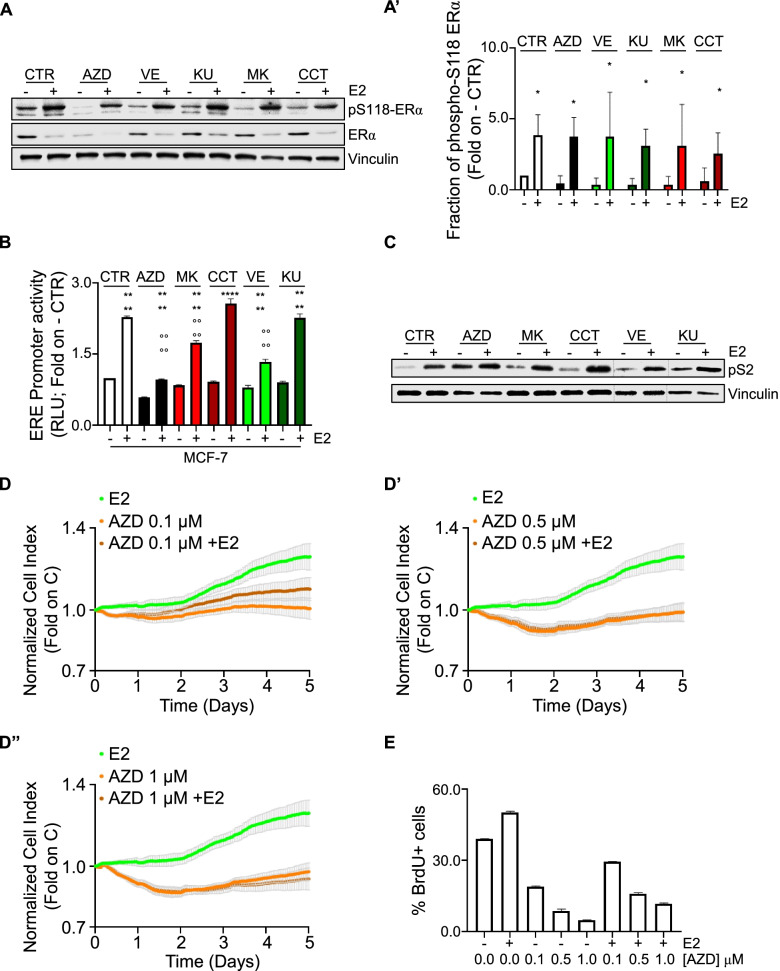
The impact of ATR:CHK1 and ATM:CHK2 pathways on E2:ERα signaling to cell proliferation. **A** Western blot of ERα and ERα S118 phosphorylation expression levels in MCF-7 pre-treated with the specific inhibitors of either CHK1 (*i.e.,* MK8776 – MK 1 μM), CHK2 (*i.e.,* CCT241533 – CCT 1 μM), ATR (*i.e.,* VE822 – VE 1 μM) or ATM (*i.e.,* KU60019 – KU 1 μM) as well as with AZD7762 (AZD 1 μM) for 24 h and then treated for 30 min with 17β-estradiol (E2 -10 nM). (**A’**) Densitometric analysis is relative to panel (**A**). The loading control was done by evaluating vinculin expression in the same filter. Panels show representative blots of three independent experiments. Significant differences with respect to - sample are calculated by Student t-test and indicated by * *p*-value < 0.05. **B** Estrogen response element promoter activity in MCF-7 ERE-NLuc cells pre-treated the specific inhibitors of either CHK1 (*i.e.,* MK8776 – MK 1 μM), CHK2 (*i.e.,* CCT241533 – CCT 0.5 μM), ATR (*i.e.,* VE822 – VE 1 μM) or ATM (*i.e.,* KU60019 – KU 1 μM) as well as with AZD7762 (AZD 1 μM) for 24 h and then treated with 17β-estradiol (E2 10 nM) for additional 24 h. The experiments were performed three times in quintuplicate duplicate. Significant differences with respect to untreated (*i.e.,* -) samples are calculated by Student t-test and indicated by **** *p*-value < 0.0001. Significant differences with respect to CTR E2 sample are calculated by Student t-test and indicated by °°°° *p*-value < 0.0001. **C** Western blot of presenilin 2 (pS2) expression levels in MCF-7 pre-treated with the specific inhibitors of either CHK1 (*i.e.,* MK8776 – MK 1 μM), CHK2 (*i.e.,* CCT241533 – CCT 0.5 μM), ATR (*i.e.,* VE822 – VE 1 μM) or ATM (*i.e.,* KU60019 – KU 1 μM) as well as with AZD7762 (AZD 1 μM) for 24 h and then treated for 24 h with 17β-estradiol (E2 -10 nM). The loading control was done by evaluating vinculin expression in the same filter. Panels show representative blots of three independent experiments. The present blot has been merged by different blots; dotted lines represents points where gel images have been merged. Histogram depicting the densitometric analysis is available in supplementary Fig. 4B. Original Western blots are provided in supplementary table 2. (**D**, **D’** and **D’’**) Real-time growth curves in MCF-7 cells treated with AZD7762 (AZD) at the indicated doses in the absence and the presence of 17β-estradiol (E2 10 nM). The graphs show the normalized cell index (*i.e.,* cell number), which is detected with the xCelligence DP device and calculated at each time point with respect to the control sample. Each sample was measured in a quadruplicate. For details, please see the material and methods section. (**E**) Bromodeoxyuridine (BrdU) incorporation assay in MCF-7 cells treated with 17β-estradiol (E2 10 nM – 24 h) after 24 h pre-treatment with AZD7762 (AZD) at the indicated doses. The experiments have been performed twice in duplicate

The ERα is a ligand-activated transcription factor, which becomes activated upon E2 binding by phosphorylation at S118 and drives the transcription of those genes containing the estrogen response element (ERE) within their promoter regions [38]. In turn, we treated MCF-7 cells with E2 after the administration of AZD, VE, KU, MK, and CCT and detected ERα phosphorylation at S118. As shown in Fig. 9A and A’, none of the tested inhibitors prevented the E2-induced increase of the S118 phosphorylated ERα fraction in MCF-7 cells.

Next, we evaluated the ERα's ability to activate a synthetic ERE-containing reporter gene stably transfected in MCF-7 cells [12] in the presence and the absence of both the above-indicated kinase inhibitors and E2. As expected [38], E2 increased the ERα transcriptional activity (Fig. 9B). The pre-treatment of MCF-7 cells with either AZD, MK, or VE significantly reduced both the basal and the E2-induced ERα-mediated activation on the ERE-containing synthetic promoter (Fig. 9B). Notably, E2 was still significantly able to increase ERα transcriptional activity in the presence of AZD, MK, and VE treatment (Fig. 9B). On the contrary, administration of either KU or CCT did not affect the E2-triggered activation of the ERα transcriptional activity (Fig. 9B). To substantiate these findings, we additionally studied the receptor transcriptional activity in the Y537S cells stably transfected with the synthetic ERE reporter gene [17] because their mutated ERα is a transcriptional hyperactive receptor variant, which assumes a constitutively active agonist structural conformation, identical to that of the wild type receptor bound to E2 [24]. Also in this model system, we observed that AZD, MK, or VE but not KU or CCT reduced in a dose dependent-manner the transcriptional activity of the Y537S ERα variant (Supplementary Fig. 4A).

Finally, the ability of E2 to regulate the expression of a classic ERE-containing gene (*i.e.,* presenilin2—pS2 also known as trefoil factor 1—TFF1) both in the presence and in the absence of AZD, VE, KU, MK, and CCT was assessed in MCF-7 cells. As shown in Fig. 9C and Supplementary Fig. 4B, none of the tested inhibitors dampened the E2-induced increase in pS2 intracellular levels.

Overall, these data indicate that the ATR:CHK1 axis inhibition decreases ERα transcriptional activity, but it does not block the ability of E2 to activate the receptor and to control gene expression.

The E2-dependent activation of ERα in BC cells results in DNA synthesis, cell cycle progression, and cell proliferation [34, 38]. In turn, we studied the effect of AZD on the E2 ability to induce cell proliferation in MCF-7 cells. As expected, E2 increased the cell number in a time-dependent manner (Fig. 9D-D’’). Co-treatment of MCF-7 cells with different doses of AZD prevented in a dose-dependent manner both the basal and the E2-induced time-dependent increase in cell number (Fig. 9D-D’’). Accordingly, AZD reduced in a dose-dependent manner the ability of E2 to increase bromodeoxyuridine (BrdU) incorporation in MCF-7 cells (Fig. 9E).

Altogether, these data indicate that inhibition of CHK1 activity interferes with the ability of E2 to induce DNA synthesis and cell proliferation in MCF-7 cells.

### Endocrine therapy drugs and CHK1 inhibitors as a novel combinatorial approach for the treatment of primary and metastatic BC

The obtained data indicate that CHK1 could be an appealing target for the treatment of ERα breast tumors [36, 37]. As noted above, CHK1 inhibitors are currently tested in clinical trials for the treatment of several solid tumors and some CHK1 inhibitors can be administered safely, but especially when combined with traditional chemotherapeutic agents, their non-transformed tissue toxicity exceeds their gains in therapeutic efficacy [36, 37]. In turn, no CHK1 inhibitor has reached phase III evaluation or FDA approval [36]. Nonetheless, it has been proposed that combinatorial treatment of CHK1 inhibitors with other modulators of proliferative signaling could allow to scale down the therapeutical CHK1 inhibitor doses, thus reducing their detrimental profiles [36]. On the other hand, ET and in particular Tam is the mainstay treatment for ERα-positive primary BC, especially the LumA ones [2, 4, 5], while CDK4/CDK6 inhibitors (*i.e.,* palbociclib—Palbo and abemaciclib—Abe) are co-adjuvant drugs for the treatment of MBC expressing the ERα [1, 2, 4]. Notably, both Tam, Palbo, and Abe are routinely used in clinical practice [1, 2, 4].

**Fig. 10:**
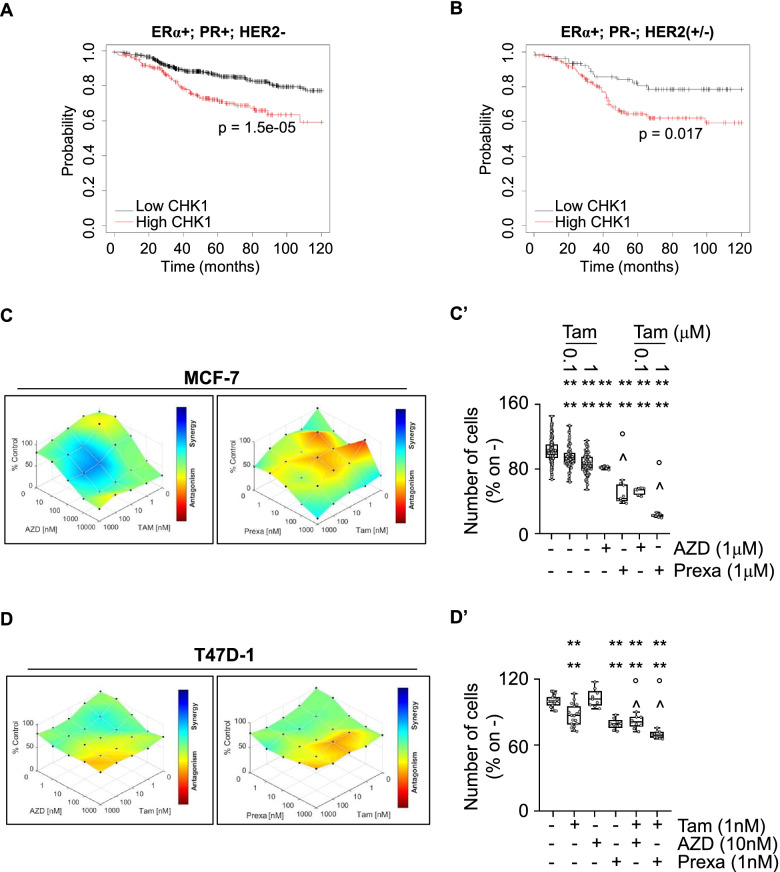
CHK1 inhibitors synergic effects with 4OH-Tamoxifen in MCF-7 cells. Kaplan–Meier plots showing the relapse-free survival (RFS) probability in women carrying breast tumors expressing either ERα, progesterone receptor (PR) but not HER2 (**A**) or ERα, but not PR or both expressing or not HER2 (**B**) as a function of CHK1 mRNA levels. All possible cutoff values between the lower and upper quartiles are automatically computed (*i.e.,* auto select best cutoff on the website), and the best performing threshold is used as a cutoff [27]. Details of the parameters of the curves are given in supplementary table 3. Significant differences between the RFS are given as *p*-value in each panel. Synergy map of 12 days-treated MCF-7 (**C**) and T47D-1 (**D**) cells with different doses of 4OH-Tamoxifen (Tam) and AZD7762 (AZD) or prexasertib (Prexa) (**C**, and **D** left and right panels, respectively). Growth curves in MCF-7 (**C’**) or T47D-1 (**D’**) cells show the synergic effect of each combination of compounds with selected doses. Significant differences with respect to untreated (*i.e.,* -,-) samples are calculated by Student t-test and indicated by **** *p*-value < 0.0001. Significant differences between Tam + AZD with respect to Tam alone or AZD alone are calculated by Student t-test and indicated by ° *p*-value < 0.05. Significant differences between Tam + Prexa with respect to Tam alone or Prexa alone are calculated by Student t-test and indicated by ^ *p*-value < 0.05. For details, please see the material and methods section

Therefore, we next decided to test if CHK1 inhibitors (*i.e.,* AZD, MK, GDC, and Prexa) could be used in combination with Tam and/or CDK4/CDK6 inhibitors in cell lines modeling primary and MBC. Analysis of RFS in patients carrying ERα-positive tumors stratified according to the LumA (*i.e.,* ERα-positive, progesterone receptor (PR)-positive, HER2-negative) or the LumB (*i.e.,* ERα-positive, PR-negative, HER2-negative/positive) [27, 42] phenotype revealed that although a significant increase in the survival probability was observed in women with BCs expressing low levels of CHK1, patients with LumA tumors appears more significantly sensitive to reduced levels of CHK1 (Fig. 10A and B). In turn, we performed proliferation studies in two LumA (*i.e.,* MCF-7 and T47D-1) and two LumB (*i.e.,* BT-474 and MDA-MB-361) BC cell lines [42] treated with different doses of either AZD, MK, GDC, and Prexa and with different doses of Tam for 12 days. Data show that synergy between Tam and AZD or Prexa was detected only in MCF-7 (Fig. 10C and C’) and T47D-1 (Fig. 10D and D’) cells but not in BT-474 and MDA-MB-361 (Supplementary Fig. 5), thus supporting the concept that these inhibitors could be used in the combinatorial treatment of LumA rather than LumB BCs. Notably, no synergic effect was identified when MCF-7 cells were treated with Tam in combination with either MK or GDC (Supplementary Fig. 5).

**Fig. 11:**
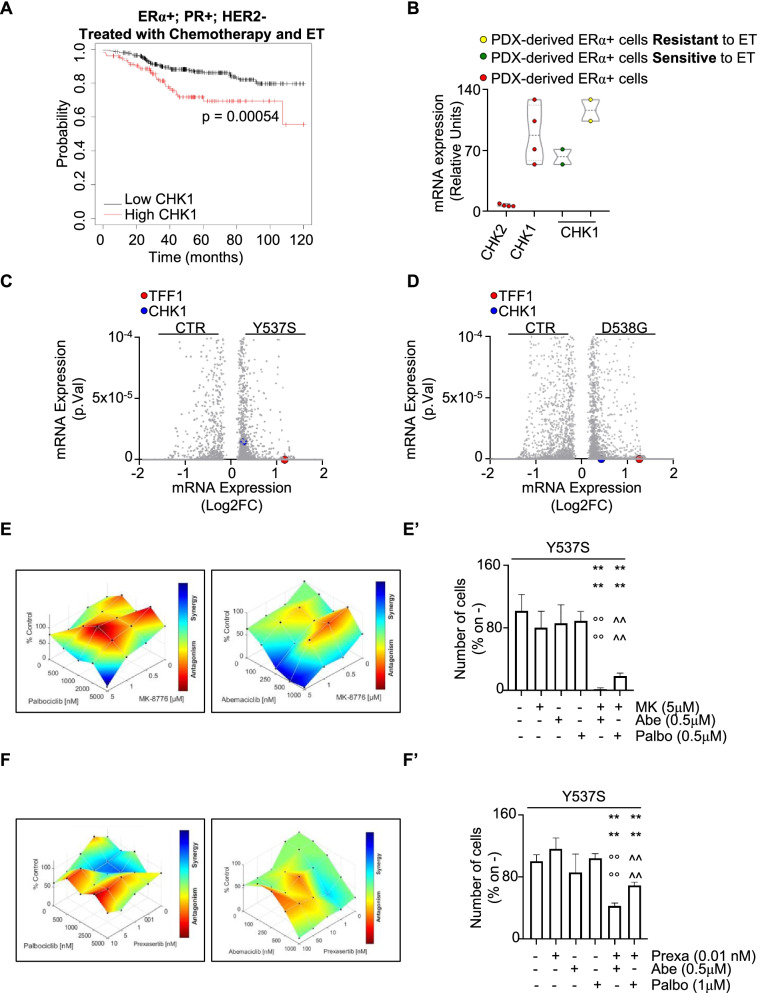
CHK1 inhibitors synergic effects with palbociclib and abemaciclib in Y537S cells. **A** Kaplan–Meier plots showing the relapse-free survival (RFS) probability in women carrying breast tumors expressing ERα, PR, or both expressing or not HER2 as a function of CHK1 mRNA levels. All possible cutoff values between the lower and upper quartiles are automatically computed (*i.e.,* auto select best cutoff on the website), and the best performing threshold is used as a cutoff [27]. Details of the parameters of the curves are given in supplementary table 3. Significant differences between the RFS are given as p-value in each panel. **B** mRNA expression levels of CHK1 and CHK2 in patient-derived xenograft (PDX) immortalized cell lines stratified as indicated in the graph. Data have been extracted by the supplementary materials given in [43] . Volcano plot showing the mRNA expression as a function of the *p*-value of the indicated proteins (*i.e.,* presenilin2 -pS2, TFF—red, CHK1 – blue) in CRISPR/CAS9 engineered MCF-7 cells expressing the Y537S (**C**) or the D538G (**D**) ERα mutant as extracted by the supplementary materials given in [44]. Synergy map of 5 days-treated Y537S cells with different doses of MK8776 (MK) (**E**) or prexasertib (Prexa) and palbociclib (Palbo) or abemaciclib (Abe) (**E** and **F** left and right panels, respectively). Significant differences with respect to untreated (*i.e.,* -,-) samples are calculated by Student t-test and indicated by **** *p*-value < 0.0001. Significant differences between MK + Abe with respect to MK alone or Abe alone are calculated by Student t-test and indicated by °°°° *p*-value < 0.0001. Significant differences between MK + Palbo with respect to MK alone or Palbo alone are calculated by Student t-test and indicated by ^^^^ *p*-value < 0.0001. Significant differences between Prexa + Abe with respect to Prexa alone or Abe alone are calculated by Student t-test and indicated by °°°° *p*-value < 0.0001. Significant differences between Prexa + Palbo with respect to Prexa alone or Palbo alone are calculated by Student t-test and indicated by ^^^^ *p*-value < 0.0001. Growth curves in Y537S cells showing the synergic effect of each combination of compounds with selected doses are shown in (**E’** and **F’**). For details, please see the material and methods section

We further tested if CHK1 inhibitors could be effective also in a metastatic context. Because a significant fraction of LumA BCs after treatment with chemotherapy and ET develop a metastatic ET resistant phenotype [2, 4], we reasoned that the evaluation of the survival probability as a function of CHK1 expression in patients carrying LumA BCs that underwent both chemotherapy and ET administration would provide clues to address this issue [27]. As shown in Fig. 11A, the RFS of such patients was significantly increased if the tumor expresses low levels of CHK1. Next, we evaluated the levels of both CHK1 and CHK2 in BC cell lines derived from patient-derived xenografts (PDX) that have been characterized for their ability to respond to ET [43]. CHK1 mRNA expression was higher than CHK2 mRNA expression in ERα-positive PDX-derived cell lines and, more interestingly, the highest CHK1 expression was detected in ERα-positive PDX-derived cell lines resistant to ET drugs (Fig. 11B). Accordingly, CHK1 mRNA expression was significantly upregulated in MCF-7 cells CRISPR/CAS9 engineered to encode for the two ERα variants (*i.e.,* Y537S and D538G) [44], which are the most frequently expressed in ET-resistant MBC [24] (Fig. 11C and D). Notably, pS2 (*i.e.,* TFF1) was upregulated in both cell lines as expected (Fig. 11C and D). Therefore, these observations together with the obtained results indicate that CHK1 could be considered also a target in BC cell lines resistant to ET.

On this basis, we next treated Y537S cells with either Abe or Palbo in combination with either AZD, MK, GDC, or Prexa for 7 days. Results show that no synergy was detected when cells were co-treated with AZD and the CDK4/CDK6 inhibitors (Supplementary Fig. 6). The proliferation of Y537S cells was synergistically reduced when GDC was co-administered with Abe (Supplementary Fig. 5A and 5A’) but not with Palbo (Supplementary Fig. 6). On the contrary, treatment of Y537S cells with MK (Fig. 11E and E’) or with Prexa (Fig. 11F and F’) in combination with either Abe or Palbo increased the anti-proliferative effects of each inhibitor.

Overall, these data demonstrate that CHK1 inhibitors selectively exert synergistic anti-proliferative activities with drugs being used for the treatment of primary and MBC.

### Evaluation of CHK1 inhibitors as anti-proliferative compounds in 3D models of primary and metastatic BC

Finally, we studied the anti-proliferative effects of CHK1 inhibitors (*i.e.,* AZD, Prexa, MK) in MCF-7 and Y537S tumor cell spheroids [7, 9] as well as in alginate-based cultures [22, 23] to understand if differences in the effect of these drugs exist in cells grown in 3D structures [45].

**Fig. 12:**
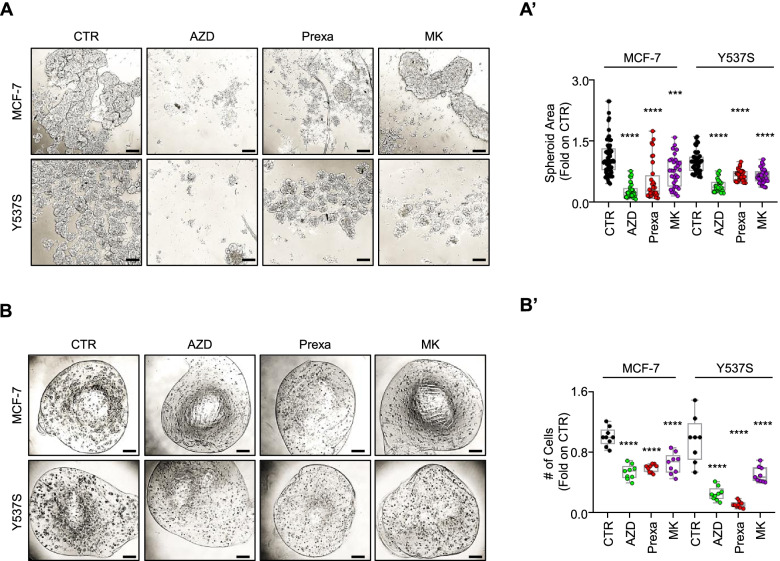
Effect of CHK1 inhibitors in 3D-models of breast cancer. Images (**A**, **B**) and quantitation (**A’**, **B’**) of tumor spheroids surface area (**A**, **A’**) and alginate-based cultures (**B**, **B’**) generated in MCF-7 and Y537S cells, treated at time 0 with CHK1 inhibitors (*i.e.,* AZD7762, AZD—1 μM; prexasertib, Prexa—1 μM; MK8776—MK 5 μM) or left untreated (CTR), for 7 days. The number of replicates is given as solid dots in the graphs. Significant differences with respect to the CTR sample were determined by unpaired two-tailed Student’s t-test: **** *p*-value < 0.0001; *** *p*-value < 0.001. Scale bars equal to 200 µm for panel **A** and 50.0 mm for panel **B**

Tumor spheroids and cells included in alginate-based spheres were counted at time 0 (*i.e.,* before drug administration), and at the end of the treatment (*i.e.,* 7 days). Both cell lines grew as spheroids and in the alginate spheres within the experimental window (*i.e.,* 7 days). All the tested inhibitors were able to significantly prevent MCF-7 and Y537S cell proliferation both as spheroid (Fig. 12A and A’) and in alginate-based cultures (Fig. 12B and B’). Therefore, CHK1 inhibitors retain their anti-proliferative activity also in a 3D environment.

### Discussion

Breast cancer (BC) is a heterogeneous disease with different molecular phenotypes. Luminal BCs express the ERα and represent about 70% of all the initial diagnoses. Luminal BCs have a favorable prognosis because the presence of the ERα dictates the clinical use of the endocrine therapy (ET) drugs (*i.e.,* aromatase inhibitors, Tam and fulvestrant), which aim to eliminate ERα and/or its signaling from BC cells [1–5].

Despite its overall efficacy, the ET still has limitations. Luminal tumors display differential sensitivity to the hormonal treatment with the drugs targeting ERα being more effective in LumA than LumB tumors. Moreover, because patient administration with ET drugs continues for 5 to 10 years after the first diagnosis, women carrying luminal BC have a very high probability (*i.e.,* about 50%) to develop resistance. Resistance to ET results in a relapse of the disease, which becomes metastatic. Increasing doses of the ET drugs are used to treat metastatic BCs (MBC) still expressing the ERα provided alone or in combination with the inhibitors of the CDK4/CDK6 kinases (*i.e.,* abemaciclib—abe; palbociclib—Palbo) but, notwithstanding these additional therapeutic approaches, MBCs remains in most cases fatal [1–5].

Therefore, there is an urgent need to identify new drugs that could avoid the generation of ET resistance in the primary tumors and/or that could be useful for the management of the metastatic disease. Recently, we decided to tackle these challenges by trying to identify molecules, which could work as ‘anti-estrogen-like’ drugs by inducing ERα degradation and killing BC cells modeling the primary and the metastatic disease. To this purpose, we set up a screening platform to measure the effect of libraries of molecules on several aspects of ERα signaling (*e.g.,* receptor stability and cell proliferation) and discovered ‘anti-estrogen-like’ activities in several FDA-approved drugs not intended for BC treatment [6–13].

Because many clinical trials are in place to test the possibility to treat luminal MBCs with drugs targeting kinases other than CDK4/CDK6 [1], we applied here our screening methods and administered a library of kinase inhibitors to ET drugs sensitive (*i.e.,* MCF-7 cells) and resistant (*i.e.,* Tam Res and Y537S MCF-7 cells) BC cell lines [14, 15] to identify kinase inhibitors working as ‘anti-estrogen-like’ drugs.

Results of the screen evidenced how most of the identified drugs inhibit enzymes belonging to the PI3K/AKT/mTOR pathway. These observations not only support the strategy to target this pathway to clinically treat luminal MBCs [1] but also reveal that drugs inhibiting this kinase cascade could induce ERα degradation. Accordingly, previous work from our laboratory indicated that administration of a PI3K inhibitor to MCF-7 cells triggers basal ERα degradation [46]. Moreover, this evidence suggests that the kinase inhibitors, which are being tested in clinical trials to prevent the growth (*i.e.,* cell proliferation) of luminal MBCs could also possess still unrecognized ‘anti-estrogen-like’ functions. This hypothesis is currently under investigation in our laboratory.

Remarkably, statistically driven selection of positive hits in our screening procedure unexpectedly identified the CHK1/CHK2 inhibitor AZD7762 (AZD) as a drug reducing ERα expression and proliferation in cells modeling primary and MBCs.

The analysis of the impact of CHK1 and CHK2 expression in BC progression revealed that the presence of each one of these two kinases is inversely correlated with the relapse-free survival (RFS) probability only in women carrying ERα-positive BCs. However, although CHK1 and CHK2 protein levels are reduced in ERα-positive tumors *versus* the ERα-negative ones, the activation status of CHK1 but not that of CHK2 is linearly correlated with the ERα activation status. Accordingly, only ERα-positive BC cell lines display an overall increased sensitivity in terms of cell survival both to the reduction of CHK1 expression and to the treatment with many different CHK1 inhibitors. Interestingly, the CHK1 and ERα activation status are even more significantly correlated in LumA BC cell lines, and women with LumA breast tumors expressing low levels of CHK1 have a significantly higher RFS probability than women with LumB breast tumors. Moreover, stratification of patients with LumA BCs treated with ET and chemotherapy indicates that reduction in CHK1 levels also prolongs their survival rate. Remarkably, the expression of CHK1 is upregulated in PDX-derived and artificially engineered ET-resistant BC cell lines [43, 44]. Accordingly, CHK1 has been additionally related to ER/PR status by other investigators [47, 48]. Consequently, we conclude that CHK1 can be considered as an appealing target for a novel treatment of ERα expressing primary and MBCs.

This assumption is further supported by the reported evaluations of the molecular links between CHK1 and ERα. Indeed, in addition to AZD, the CHK1 inhibitors tested in this work (*i.e.,* MK, Prexa, and GDC-0575—GDC) induce ERα degradation and prevent the proliferation of ET-resistant and ET-sensitive BC cell lines. In this respect, it is worth stressing that these drugs also induce the degradation of the Y537S receptor variant, which is resistant to both classical and novel selective ERα degraders and is the most common ERα point mutant found in MBC patients that developed resistance to ET drugs [15, 24]. Many ERα point mutants have been identified in MBCs in addition to the Y537S one and investigations are in place to uncover drugs that selectively eliminate those receptor mutants from cells [49, 50]. Present results suggest that CHK1 inhibitors are an addition to the repertoire of such compounds.

CHK1 and CHK2 are the downstream effector of ATR and ATM, respectively, and the ATR:CHK1 and ATM:CHK2 pathways are mainly involved in the control of genome integrity as their activities are required to buffer DNA damages and to sustain cell proliferation [34, 39, 51]. We report for the first time that the ATR:CHK1 pathway but not the ATM:CHK2 pathway activity is required to maintain ERα stability and expression. In turn, ERα-positive BC cell lines are more sensitive to the anti-proliferative effects of ATR and CHK1 inhibitors rather than those elicited by the ATM and CHK2 inhibitors. Although we did not study the mechanisms by which the inhibition of the ATM:CHK2 pathway leads to a reduction in cell proliferation, the fact that the inhibition of ATR:CHK1 axis induces receptor degradation can account for the increased dependency of the ERα-positive BC cell lines to the ATR:CHK1 pathway inhibition. Accordingly, ATR and CHK1 but not ATM and CHK2 are ‘Achille’ genes (*i.e.,* the reduction of their expression prevents cell proliferation) in luminal BC cells [32].

The analysis of the mechanism through which the ATR:CHK1 pathway controls ERα stability reveals a novel relationship between the replication stress and receptor degradation. Indeed, we observed in both MCF-7 and Y537S cells that the inhibition of ATR or CHK1 causes ERα degradation in parallel with the appearance of both replication stress and DNA damage (measured by RPA2 and γH2AX, respectively). Interestingly, the use of several inducers of replication stress (*i.e.,* ETO, CPT, Aph, HU) [41] and direct inducers of physical DNA damage (*i.e.,* X-rays) indicates that when ERα-expressing cells undergo replication stress, the receptor is eliminated. Therefore, we conclude that the administration of CHK1 inhibitors determines an increase in replication stress, which in turn results in the degradation of the ERα.

The fact that BC cells trigger receptor degradation when replication stress is induced suggests that ERα could be considered as a sensor protein for genomic stress. Moreover, because E2 induces replication stress via ERα and determines receptor degradation [34, 46, 52], it is tempting to speculate not only that replication stress is a signaling intermediate in the transduction mechanisms of E2 intracellular action but also that replication stress-dependent ERα degradation occurs to eliminate additional sources of ERα-induced replication stress, in a negative feed-back loop preserving genome integrity. A detailed characterization of the molecular steps leading from the generation of replication stress to ERα degradation also as a function of cell cycle progression is currently in progress in our laboratory.

The study of the impact of the ATR:CHK1 pathway inhibitors in E2:ERα signaling demonstrates that while E2 maintains its ability in activating ERα-dependent gene expression, the extent of the E2 effects in activating ERα transcriptional activity, cell cycle progression, and cell proliferation are strongly reduced. Thus, the control of ERα stability exerted by the ATR:CHK1 pathway does not directly implicate the modification of the functional mechanisms of the E2:ERα signaling pathway but rather it only influences the receptor intracellular abundance leading to overall reduced responsiveness to E2 with respect to transcriptional activity and cell proliferation. Thus, because the ATR:CHK1 pathway control ERα stability rather than regulating the E2-dependent ERα-mediated effects, the ATR:CHK1 pathway appears to work in parallel with the E2:ERα transduction network.

The concomitant targeting of two parallel pathways regulating cell proliferation can result in a synergistic action of the administered drugs [53]. Indeed, CHK1 inhibitors synergistically prevent ERα expressing BC cell proliferation when administered in combination with either Tam or CDK4/CDK6 inhibitors. Due to their toxicity, to date, no CHK1 inhibitor has reached FDA approval for the clinical use against solid tumors [36, 37]. Consequently, searching for the possible combinatorial application of CHK1 inhibitors with other anti-proliferative agents is strongly encouraged to scale down their treatment dosage and reduce their negative side effects [36]. Here, we report for the first time that the synergic reduction of LumA BC cell proliferation occurs with both CHK1 inhibitors, Tam, and CDK4/CDK6 inhibitors, thus suggesting that this combinatorial strategy could be effective in the treatment of ERα-positive BC sufferers. Furthermore, because different combinations of drugs (*i.e.,* AZD and Prexa but not GDC with Tam only in LumA BC cell lines; MK, GDC, and Prexa with Abe and MK and Prexa with Palbo in ET-resistant LumA cells) have synergistic effects in different cell lines, our analyses unveil a potential for personalization of co-treatments with CHK1 inhibitors in different kind of ERα-positive BC cell lines. Accordingly, recent evidence suggests that in triple negative BCs CHK1 inhibition enhances adriamycin (ADR) chemosensitivity while in LumA tumors CHK1 inhibition is not able to synergize with ADR [47, 48]. Altogether this evidence strongly supports the concept that CHK1 inhibition as a function of the different molecular characteristics of BC [4] could be used as a specific patient-driven treatment protocol.

Noteworthy, the tested inhibitors retain their anti-proliferative activities also in 3D models of BC. In this respect, although we used different 3D models as a strategy to replace animal experimentation and to study the effects of CHK1 inhibitors in a spatial environment that is closer to the situation where the drugs work in human tissues [45], the possibility to combine ET drugs and CHK1 inhibitors for the effective treatment of ERα-positive primary and metastatic tumors remains to be demonstrated in patients enrolled in specific clinical trials also because the cell lines (*e.g.,* MCF-7, Tam Res and Y537S) used in this work are *in vitro* model systems and could not completely recapitulate the complexity of the tumors *in vivo*. Nonetheless, the present study has been performed on several different cell lines widely used to model ERα-positive primary and metastatic BC that have been treated with different CHK1 inhibitors and with the drugs used to treat women carrying primary (*i.e.,* Tam) or metastatic (*i.e.,* palbociclib and abemaciclib) breast tumors. Therefore, the obtained results strongly suggest new therapeutic options for patients suffering from ERα-positive BC.

### Conclusions

In this work, we report for the first time CHK1 as a novel target for ERα-positive BC treatment. In addition, the data shown here disclose a new mechanism through which BC cells control ERα stability and abundance via CHK1 activity and further demonstrate that targeting ERα-positive cells modeling primary and MBC with clinically relevant CHK1 inhibitors alone or in combination with ET drugs (*i.e.,* Tam) or with drugs used for the treatment of MBC (*i.e.,* Abe and Palbo) represent an appealing strategy to prevent ERα-positive BC cell proliferation.

Overall, we propose that small molecule-dependent inhibition of CHK1 could either avoid the occurrence of ET resistance in luminal BC or be effective in the management of luminal MBCs.

## Supplementary Information

**Additional file 1.** **Figure 1. **Controls for AZD7762 inhibitor in MCF-7 and Y537S cells. Western blot analyses of S296 phosphorylated CHK1, S516 phosphorylated CHK2, CHK1, and CHK2 expression levels in (A) MCF-7 and (B) Y537S cells treated for 2 hours with etoposide (ETO) (10 μM) both in the presence or in the absence of 1 μM AZD7762 (AZD). The loading control was done by evaluating vinculin expression in the same filter. (C, D) Western blot analyses of CHK1 and CHK2 intracellular levels after siRNA transfection procedure. These blots are the controls for the experiments described in Fig. 4O and 4P. The loading control was done by evaluating vinculin expression in the same filter. **Figure 2. **Controls for ATR, ATM, CHK1, and CHK2 inhibitors in MCF-7 and Y537S cells. Western blot analyses in MCF-7 (A-D) and Y537S (E-H) cells treated with the indicated doses of for 24 hours with the indicated doses of the specific inhibitors of either CHK1 (*i.e.,* MK8776 - MK) (D and H), CHK2 (*i.e.,* CCT241533 - CCT) (C and G), ATR (*i.e.,* VE822 - VE) (B and F) or ATM (*i.e.,* KU60019 - KU) (A and E) both in the presence and in the absence of etoposide (ETO – 10 µM 2 hours) of the phosphorylated forms of CHK1 and CHK2. Total CHK1 and CHK2 as well as vinculin expression was evaluated as loading controls in the same filters. The experiments were performed twice. Densitometric analyses are available upon request.** Figure 3. **Impact of clinically relevant CHK1 inhibitor in inducing ERα degradation and preventing proliferation in MCF-7 and Y537S cells. Western blot (A) and relative densitometric analyses (A’) of ERα expression levels in MCF-7 (yellow) and Y537S (red) cells treated for 24 hours with the indicated doses of GDC-0575 (GDC). The loading control was done by evaluating vinculin expression in the same filter. Significant differences with respect to control (0) were obtained by unpaired two-tailed Student’s t-test. Data show the mean ± the standard deviations, **** *p* < 0.0001; ** *p* < 0.01; * *p* < 0.05. The number of replicates is given as solid dots in the bar graph. Growth curve analyses in MCF-7 (B) and Y537S (C) cells were performed as indicated in the material and method section for 5 days with different doses of GDC-0575 (GDC). The graph shows the normalized cell index (*i.e.,* cell number), which is detected with the xCelligence DP device and calculated at each time point with respect to the control sample. Each sample was measured in a quadruplicate. For details, please see the material and methods section.** Figure 4. **The impact of ATR:CHK1 and ATM:CHK2 pathways on hyperactive ERα transcriptional activity. (A) Estrogen response element promoter activity in Y537S ERE-NLuc cells pre-treated the specific inhibitors of either CHK1 (*i.e.,* MK8776 – MK 1 μM), CHK2 (*i.e.,* CCT241533 – CCT 0.5 μM), ATR (*i.e.,* VE822 – VE 1 μM) or ATM (*i.e.,* KU60019 – KU 1 μM) as well as with AZD7762 (AZD 1 μM) for 24 hours at the indicated doses. The experiments were performed three times in quintuplicate duplicate. Significant differences with respect to control (CTR) sample are calculated by Student t-test and indicated by **** *p*-value < 0.0001 and ** *p*-value < 0.01. (B) Western blot analyses of pS2 levels treated with the indicated inhibitors as described in figure 9. This histogram represents the densitometric analyses of the blots indicated in Fig. 9C.** Figure 5. **GDC-0575 synergic effects with abemaciclib in Y537S cells and synergy studies in BC cell lines. Synergy map of 5 days-treated Y537S cells with different doses of GDC-0575 (GDC) and abemaciclib (Abe) (A). Growth curves in Y537S cells showing the synergic effect of each combination of compounds with selected doses are shown in (A’). For details, please see the material and methods section. Significant differences with respect to untreated (*i.e.,* -,-) samples are calculated by Student t-test and indicated by **** *p*-value < 0.0001. Significant differences between GDC+Abe with respect to GDC alone or Abe alone are calculated by Student t-test and indicated by ° or ^ *p*-value < 0.05, respectively. Number of MCF-7 (B, B’), BT-474 (C, C’) and MDA-MB-361 (D, D’) cells treated for 12 days with the indicated doses of 4OH-Tamoxifen (Tam) in combination with the indicated doses of MK8776 (MK) (B), GDC-0575 (GDC) (B’), AZD7762 (AZD) (C, D) or prexasertib (Prexa) (C’, D’). **Figure 6.** Synergy studies in Y537S cells. The number of Y537S cells treated for 5 days with the indicated doses of AZD7762 (AZD) (A, B) or GDC-0575 (GDC) (C) in combination with the indicated doses of abemaciclib (Abe) (A) or palbociclib (Palbo) (B, C). **Figure 7. **Inhibitor-dependent effect on DNA damage and replication stress and replication stress and X-rays dependent effect on ERα levels in MCF-7 and Y537S cells. (A, B, C, E, F, and H) Histograms relative to the heatmaps shown in main Fig. 7G and 7H for the densitometric analyses of ERα (A, E), RPA2 (B, F) and phosphorylated γH2AX (C, G) expression levels in MCF-7 (A, B, and C) and Y537S (E, F, and G) cells. Description of the treatments have been given in the figure caption of the main Fig. 7. (D and H) Histograms relative to the heatmaps shown in main Fig. 8C and 8F for the densitometric analyses of ERα, RPA2 and phosphorylated γH2AX expression levels in MCF-7 (D) and Y537S (H) cells. Description of the treatments has been given in the figure caption of the main Fig. 8.

**Additional file 2. **Supplementary Table 1

**Additional file 3**. Supplementary Table 2

**Additional file 4**. Supplementary Table 3

**Additional file 5.** Supplementary Table 4

## Data Availability

All the original Western blots are available in supplementary table 2. All the Kaplan–Meier curves were retrieved by the Kaplan–Meier Plotter database and given in supplementary table 3 as downloaded by the website (https://kmplot.com/analysis/) [27]. All the datasets used to generate Fig. 2 (except Fig. 2A, please see below) and Fig. 5A and A’ were downloaded by the Broad Institute through the DepMap portal (https://depmap.org/portal) and are available in supplementary table 1. The original numbers in Fig. 11B are available in supplementary table 4 and have been extracted by the supplementary materials given in [43]. The datasets for Fig. 2A have been downloaded by the breast cancer landscape website (https://www.breastcancerlandscape.org/) as associated with the paper published in [3] while the datasets used to generate Fig. 11C and D are given in the supplementary files of the publication in [44].

## Abbreviations

Abe: Abemaciclib
AKT: V-akt murine thymoma viral oncogene homolog 1 (AKT)
Aph: Aphidicolin
ATM: Ataxia Telangiectasia Mutated
ATR: Ataxia Telangiectasia and Rad3-Related Protein
AZD: AZD7762
BC: Breast cancer
BrdU: Bromodeoxyuridine
CCT: CCT241533
CDK4: Cyclin-dependent kinase 4
CDK6: Cyclin-dependent kinase 6
CHK1: Checkpoint Kinase 1
CHK2: Checkpoint Kinase 2
CHX: Cycloheximide
CPT: Camptothecin
DMEM: Dulbecco’s Midified Eagle Medium
E2: 17β-Estradiol
ERBB2: V-Erb-B2 Avian Erythroblastic Leukemia Viral Oncogene Homolog 2
ERE: Estrogen responsive element
ERα: Estrogen receptor α
ET: Endocrine therapy
ETO: Etoposide
FDA: Food and Drug Administration
FGF-R: Fibroblast Growth Factor Receptor 1
GDC: GDC-0575
Gy: Gray
HER2: Human Epidermal Growth Factor Receptor 2
HU: Hydroxyurea
IGF1-R: Insulin Like Growth Factor 1 Receptor
KU: KU60019
LumA: Luminal A
LumB: Luminal B
MBC: Metastatic breast cancer
MET: MET Proto-Oncogene, Receptor Tyrosine Kinase
MK: MK8776
mTOR: Mammalian target of rapamycin
Palbo: Palbociclib
PDX: Patient-derived xenografts
PI: Propidium Iodide
PI3K: Phosphatidyl-inositol-3-kinase
PR: Progesterone receptor
Prexa: Prexasertib
pS2: Presenilin2
RFS: Relapse free survival
RPA2: Replication Protein A 2
Tam: 4OH-tamoxifen
TFF1: Trefoil factor 1
VE: VE822
YY: Buffer: Yoss Yarden Buffer
γH2AX: Phosphorylated H2A Histone Family Member X
