# Supplementary material for: Clinically relevant CHK1 inhibitors abrogate wild-type and Y537S mutant ERα expression and proliferation in luminal primary and metastatic breast cancer cells
Source: J Exp Clin Cancer Res. 2022 Apr 13;41:141. doi: 10.1186/s13046-022-02360-y (PMC9006609; doi:10.1186/s13046-022-02360-y)
Supplement: Supplementary file 1 — Additional file 1. Figure 1. Controls for AZD7762 inhibitor in MCF-7 and Y537S cells. Western blot analyses of S296 phosphorylated CHK1, S516 phosphorylated CHK2, CHK1, and CHK2 expression levels in (A) MCF-7 and (B) Y537S cells treated for 2 hours with etoposide (ETO) (10 μM) both in the presence or in the absence of 1 μM AZD7762 (AZD). The loading control was done by evaluating vinculin expression in the same filter. (C, D) Western blot analyses of CHK1 and CHK2 intracellular levels after siRNA transfection procedure. These blots are the controls for the experiments described in Fig. 4O and 4P. The loading control was done by evaluating vinculin expression in the same filter. Figure 2. Controls for ATR, ATM, CHK1, and CHK2 inhibitors in MCF-7 and Y537S cells. Western blot analyses in MCF-7 (A-D) and Y537S (E-H) cells treated with the indicated doses of for 24 hours with the indicated doses of the specific inhibitors of either CHK1 (i.e., MK8776 - MK) (D and H), CHK2 (i.e., CCT241533 - CCT) (C and G), ATR (i.e., VE822 - VE) (B and F) or ATM (i.e., KU60019 - KU) (A and E) both in the presence and in the absence of etoposide (ETO – 10 µM 2 hours) of the phosphorylated forms of CHK1 and CHK2. Total CHK1 and CHK2 as well as vinculin expression was evaluated as loading controls in the same filters. The experiments were performed twice. Densitometric analyses are available upon request. Figure 3. Impact of clinically relevant CHK1 inhibitor in inducing ERα degradation and preventing proliferation in MCF-7 and Y537S cells. Western blot (A) and relative densitometric analyses (A’) of ERα expression levels in MCF-7 (yellow) and Y537S (red) cells treated for 24 hours with the indicated doses of GDC-0575 (GDC). The loading control was done by evaluating vinculin expression in the same filter. Significant differences with respect to control (0) were obtained by unpaired two-tailed Student’s t-test. Data show the mean ± the standard deviations, **** p < 0.0001; ** p < 0.01; * p [file 13046_2022_2360_MOESM1_ESM.pptx]

## Slide 1
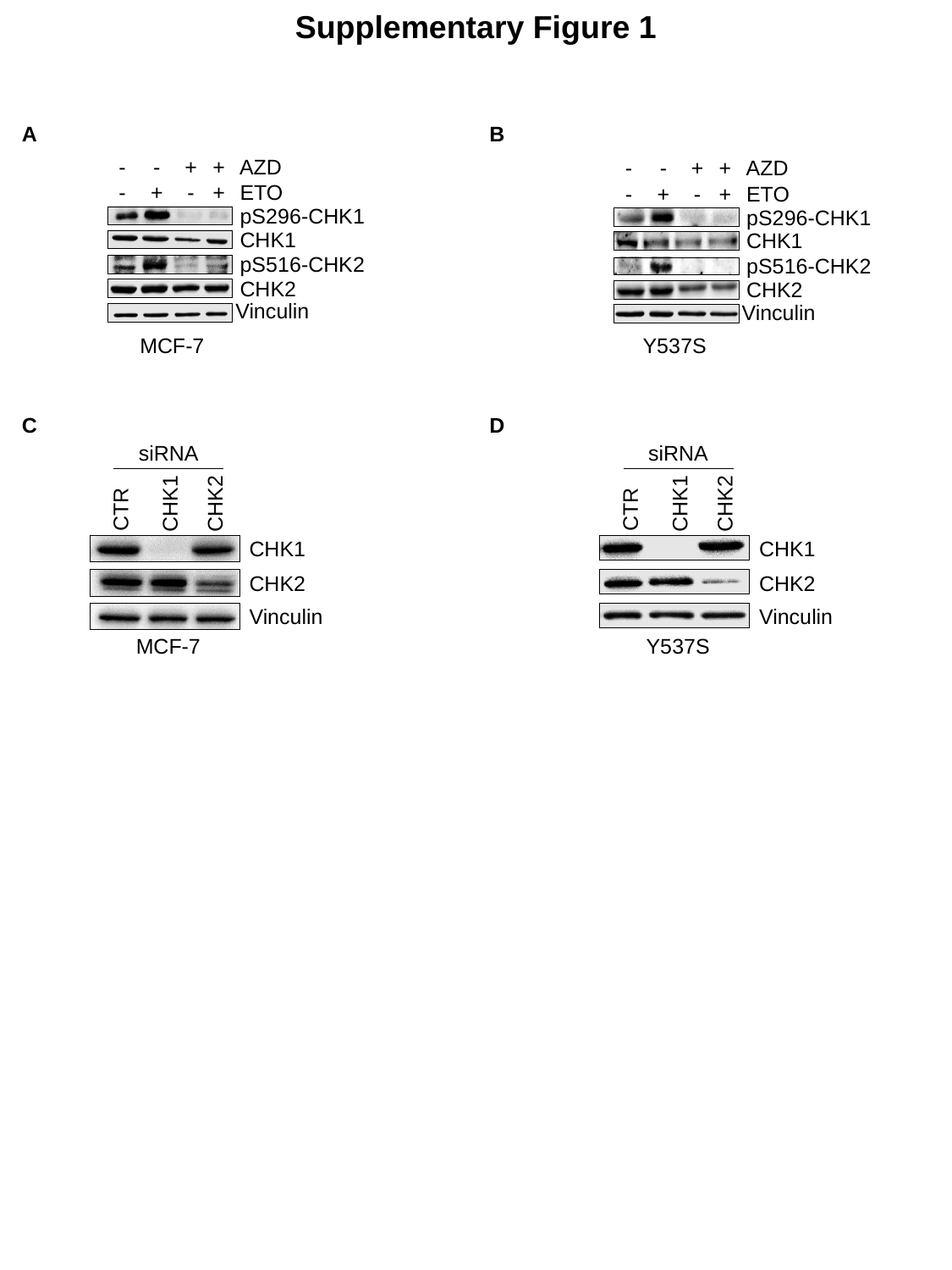

Supplementary Figure 1
A
B
-
-
+
+
AZD
-
+
-
+
ETO
pS296-CHK1
CHK1
pS516-CHK2
CHK2
Vinculin
MCF-7
-
-
+
+
AZD
-
+
-
+
ETO
pS296-CHK1
CHK1
pS516-CHK2
CHK2
Vinculin
Y537S
C
D
siRNA
CHK1
CHK2
CTR
CHK1
CHK2
Vinculin
MCF-7
siRNA
CHK1
CHK2
CTR
CHK1
CHK2
Vinculin
Y537S

## Slide 2
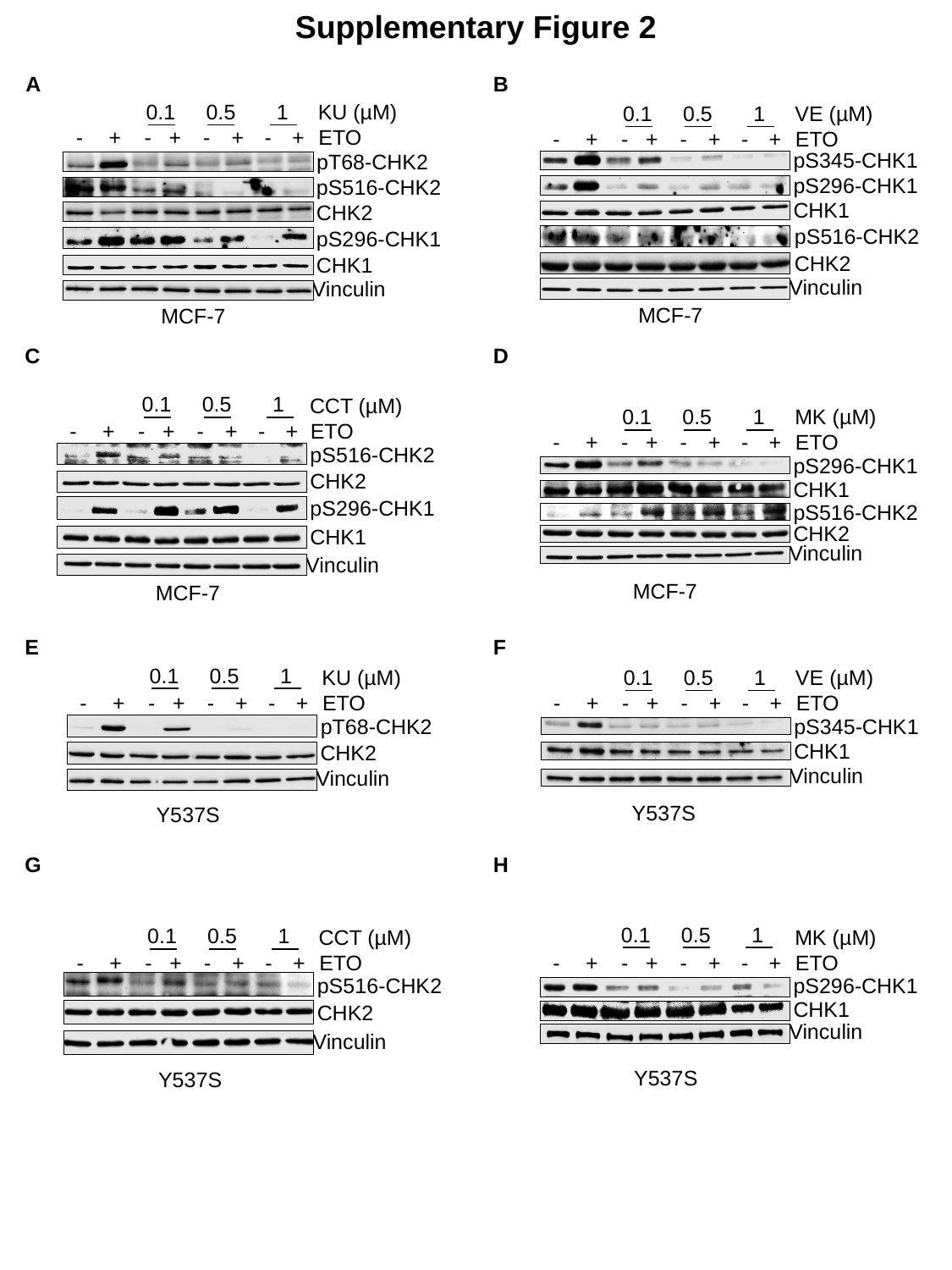

Supplementary Figure 2
B
A
0.1
0.5
1
KU (µM)
-
+
-
+
-
+
-
+
ETO
pT68-CHK2
pS516-CHK2
CHK2
pS296-CHK1
CHK1
Vinculin
MCF-7
0.1
0.5
1
VE (µM)
-
+
-
+
-
+
-
+
ETO
pS345-CHK1
pS296-CHK1
CHK1
pS516-CHK2
CHK2
Vinculin
MCF-7
C
D
0.1
0.5
1
CCT (µM)
-
+
-
+
-
+
-
+
ETO
pS516-CHK2
CHK2
pS296-CHK1
CHK1
Vinculin
MCF-7
MK (µM)
0.1
0.5
1
-
+
-
+
-
+
-
+
ETO
pS296-CHK1
CHK1
pS516-CHK2
CHK2
Vinculin
MCF-7
E
F
0.1
0.5
1
KU (µM)
-
+
-
+
-
+
-
+
ETO
pT68-CHK2
CHK2
Vinculin
Y537S
0.1
0.5
1
VE (µM)
-
+
-
+
-
+
-
+
ETO
pS345-CHK1
CHK1
Vinculin
Y537S
G
H
0.1
0.5
1
MK (µM)
-
+
-
+
-
+
-
+
ETO
pS296-CHK1
CHK1
Vinculin
Y537S
0.1
0.5
1
CCT (µM)
-
+
-
+
-
+
-
+
ETO
pS516-CHK2
CHK2
Vinculin
Y537S

## Slide 3
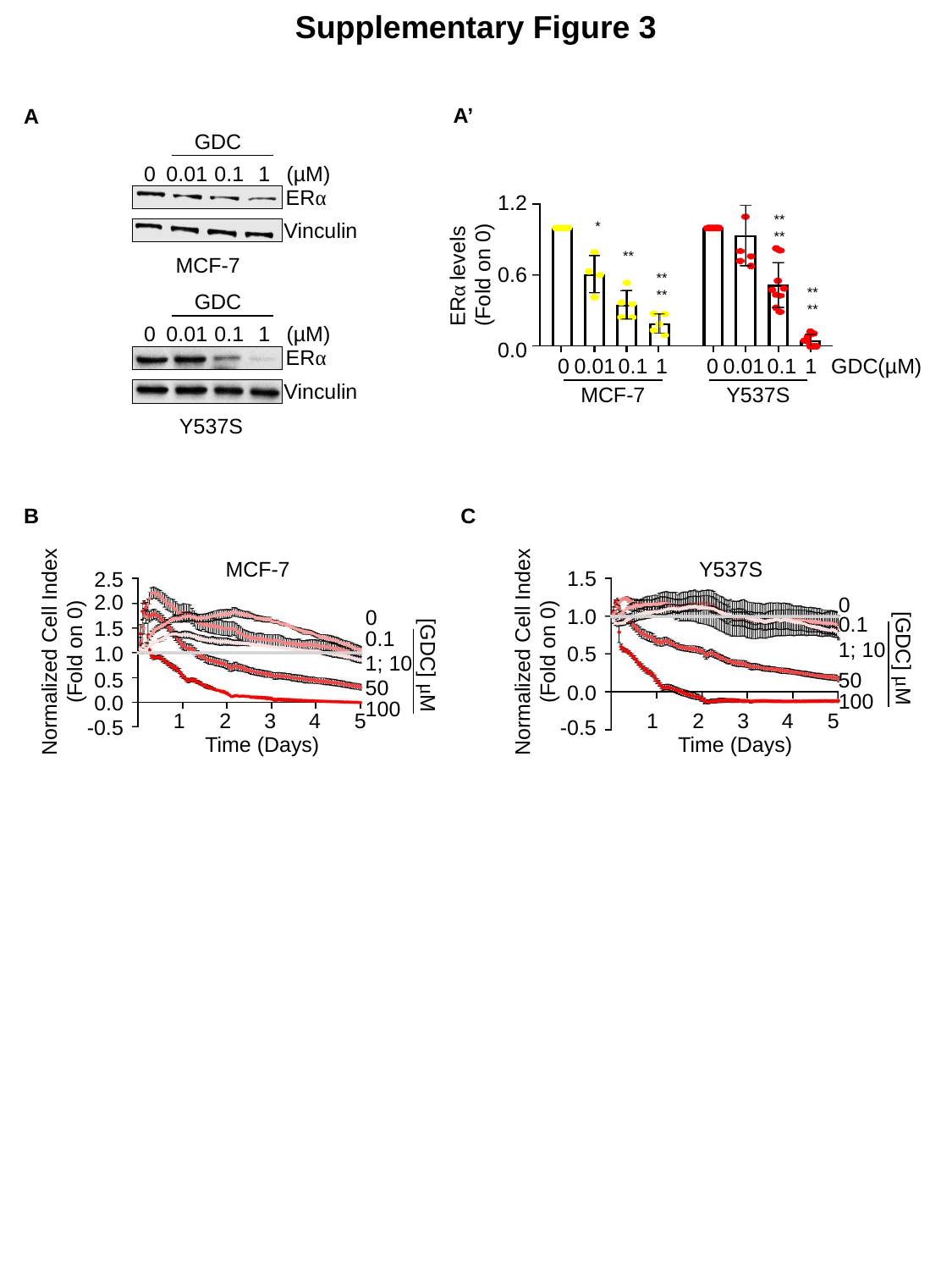

Supplementary Figure 3
A’
A
GDC
0
0.01
0.1
1
 (µM)
ERα
Vinculin
MCF-7
1.2
**
**
*
**
ERα levels
(Fold on 0)
0.6
**
**
**
**
0.0
0
0.01
0.1
1
0
0.01
0.1
1
GDC(µM)
MCF-7
Y537S
GDC
0
0.01
0.1
1
 (µM)
ERα
Vinculin
Y537S
B
C
Y537S
1.5
0
1.0
0.1
1; 10
Normalized Cell Index
(Fold on 0)
0.5
[GDC] μM
50
0.0
100
1
2
3
4
5
 Time (Days)
-0.5
MCF-7
2.5
2.0
0
1.5
0.1
1; 10
Normalized Cell Index
(Fold on 0)
1.0
[GDC] μM
0.5
50
0.0
100
1
2
3
4
5
 Time (Days)
-0.5

## Slide 4
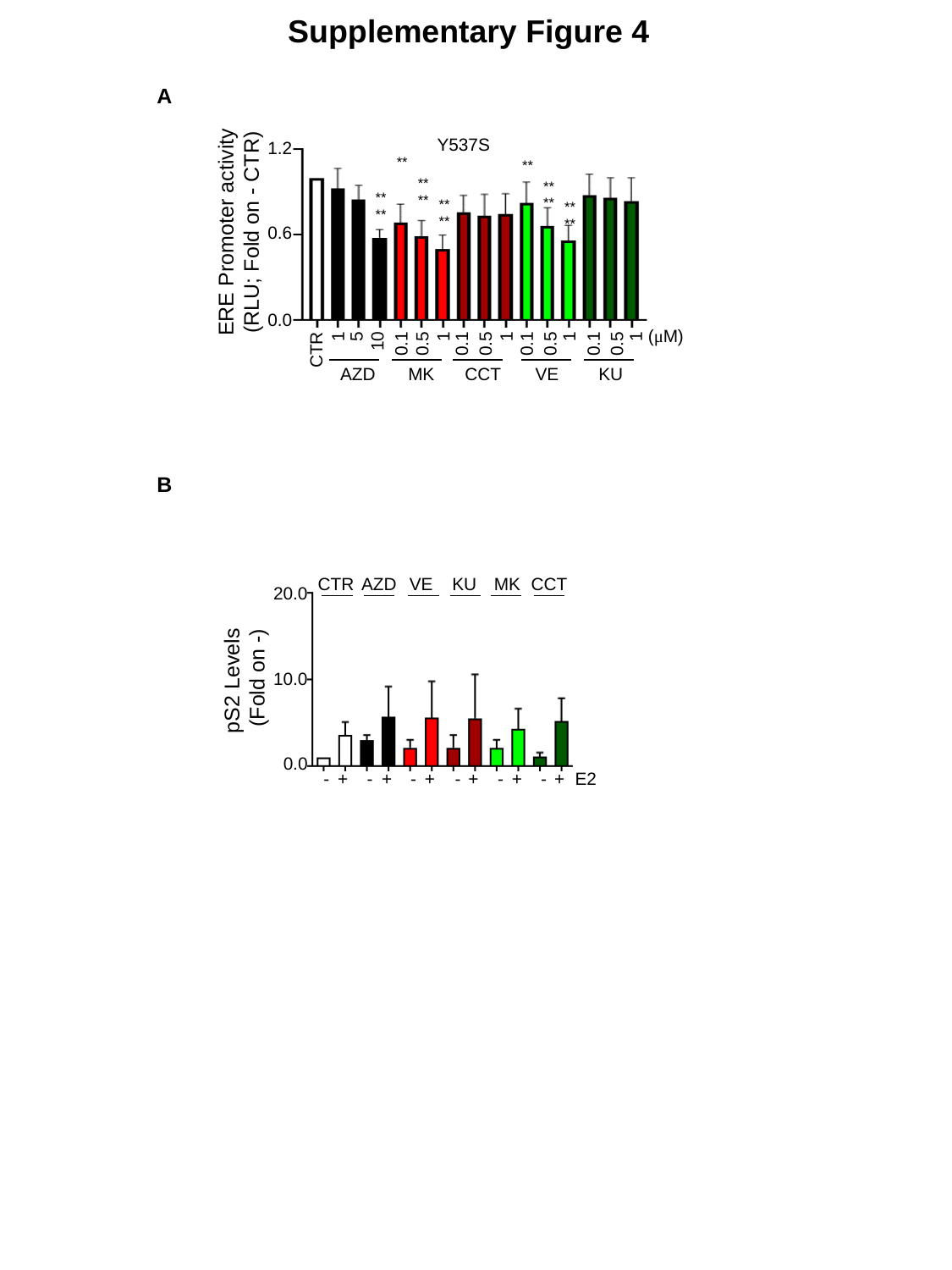

Supplementary Figure 4
A
1.2
ERE Promoter activity
(RLU; Fold on - CTR)
0.6
0.0
(μM)
1
5
1
1
1
1
10
0.1
0.5
0.1
0.5
0.1
0.5
0.1
0.5
CTR
AZD
MK
CCT
VE
KU
**
**
**
**
**
**
**
**
**
**
**
**
Y537S
B
CTR
AZD
VE
KU
MK
CCT
20.0
pS2 Levels
(Fold on -)
10.0
0.0
-
+
-
+
-
+
-
+
-
+
-
+
E2

## Slide 5
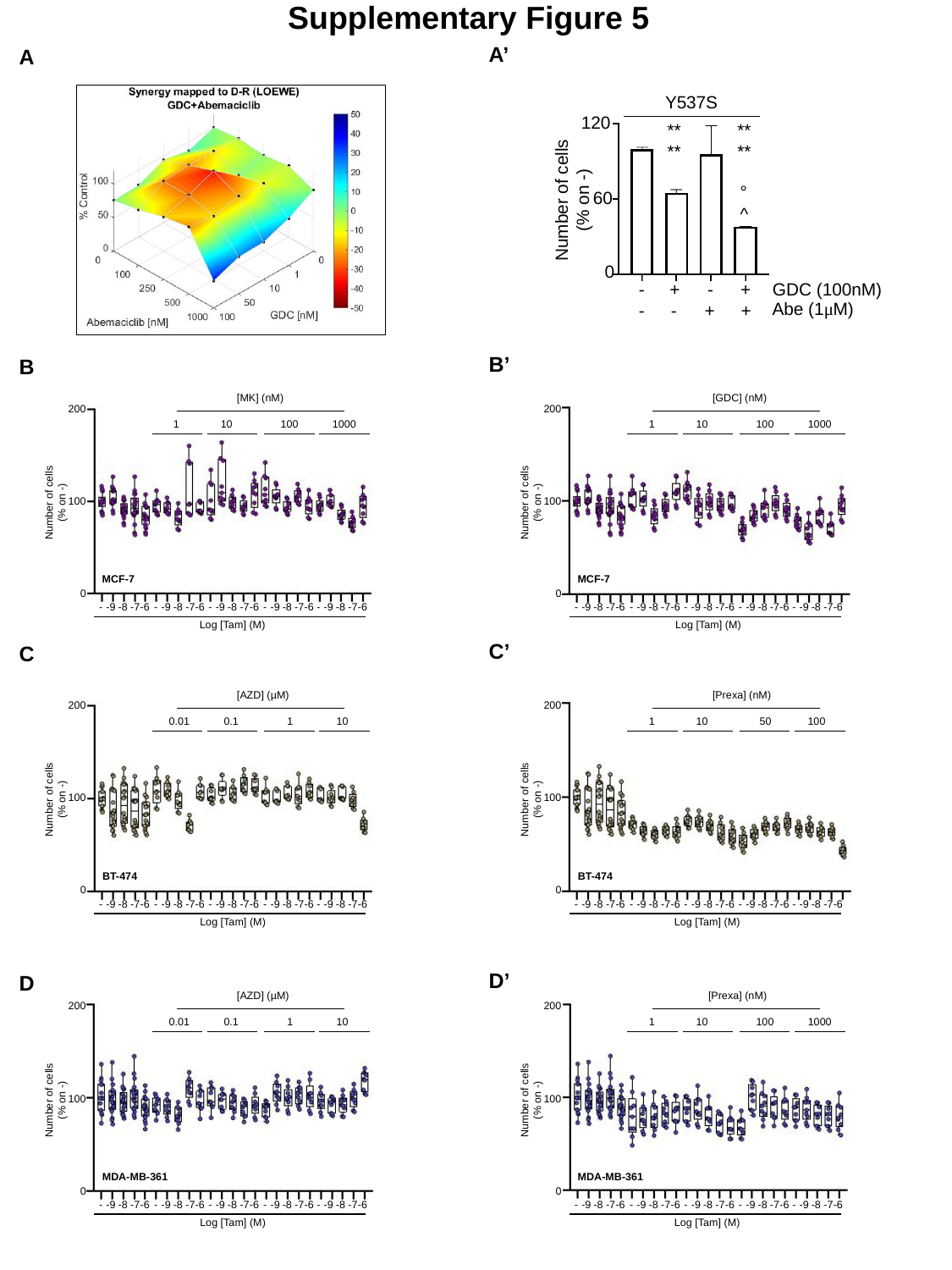

Supplementary Figure 5
A’
A
Y537S
120
**
**
**
**
Number of cells
(% on -)
°
^
60
0
-
+
-
+
GDC (100nM)
Abe (1μM)
-
-
+
+
B’
B
[MK] (nM)
200
1
10
100
1000
Number of cells
(% on -)
100
MCF-7
0
-
-9
-8
-7
-6
-
-9
-8
-7
-6
-
-9
-8
-7
-6
-
-9
-8
-7
-6
-
-9
-8
-7
-6
Log [Tam] (M)
[GDC] (nM)
200
1
10
100
1000
100
MCF-7
0
-
-9
-8
-7
-6
-
-9
-8
-7
-6
-
-9
-8
-7
-6
-
-9
-8
-7
-6
-
-9
-8
-7
-6
Log [Tam] (M)
Number of cells
(% on -)
C’
C
[AZD] (µM)
200
0.01
0.1
1
10
Number of cells
(% on -)
100
BT-474
0
-
-9
-8
-7
-6
-
-9
-8
-7
-6
-
-9
-8
-7
-6
-
-9
-8
-7
-6
-
-9
-8
-7
-6
Log [Tam] (M)
[Prexa] (nM)
200
1
10
50
100
Number of cells
(% on -)
100
BT-474
0
-
-9
-8
-7
-6
-
-9
-8
-7
-6
-
-9
-8
-7
-6
-
-9
-8
-7
-6
-
-9
-8
-7
-6
Log [Tam] (M)
D’
D
[AZD] (µM)
200
0.01
0.1
1
10
Number of cells
(% on -)
100
MDA-MB-361
0
-
-9
-8
-7
-6
-
-9
-8
-7
-6
-
-9
-8
-7
-6
-
-9
-8
-7
-6
-
-9
-8
-7
-6
Log [Tam] (M)
[Prexa] (nM)
200
1
10
100
1000
Number of cells
(% on -)
100
MDA-MB-361
0
-
-9
-8
-7
-6
-
-9
-8
-7
-6
-
-9
-8
-7
-6
-
-9
-8
-7
-6
-
-9
-8
-7
-6
Log [Tam] (M)

## Slide 6
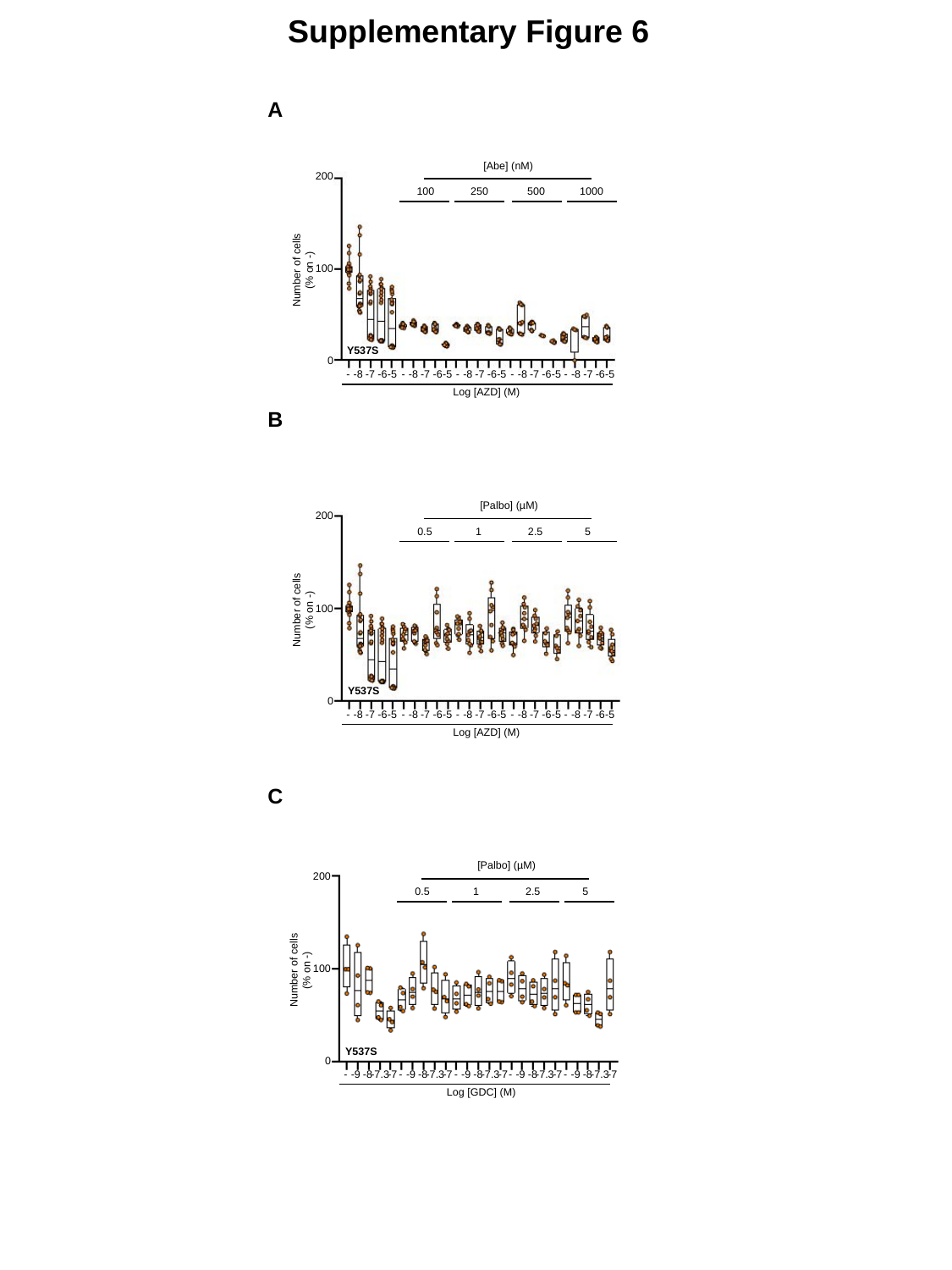

Supplementary Figure 6
A
[Abe] (nM)
200
100
250
500
1000
Number of cells
(% on -)
100
Y537S
0
-
-8
-7
-6
-5
-
-8
-7
-6
-5
-
-8
-7
-6
-5
-
-8
-7
-6
-5
-
-8
-7
-6
-5
Log [AZD] (M)
B
[Palbo] (µM)
200
0.5
1
2.5
5
Number of cells
(% on -)
100
Y537S
0
-
-8
-7
-6
-5
-
-8
-7
-6
-5
-
-8
-7
-6
-5
-
-8
-7
-6
-5
-
-8
-7
-6
-5
Log [AZD] (M)
C
[Palbo] (µM)
200
0.5
1
2.5
5
Number of cells
(% on -)
100
Y537S
0
-
-9
-8
-7.3
-7
-
-9
-8
-7.3
-7
-
-9
-8
-7.3
-7
-
-9
-8
-7.3
-7
-
-9
-8
-7.3
-7
Log [GDC] (M)

## Slide 7
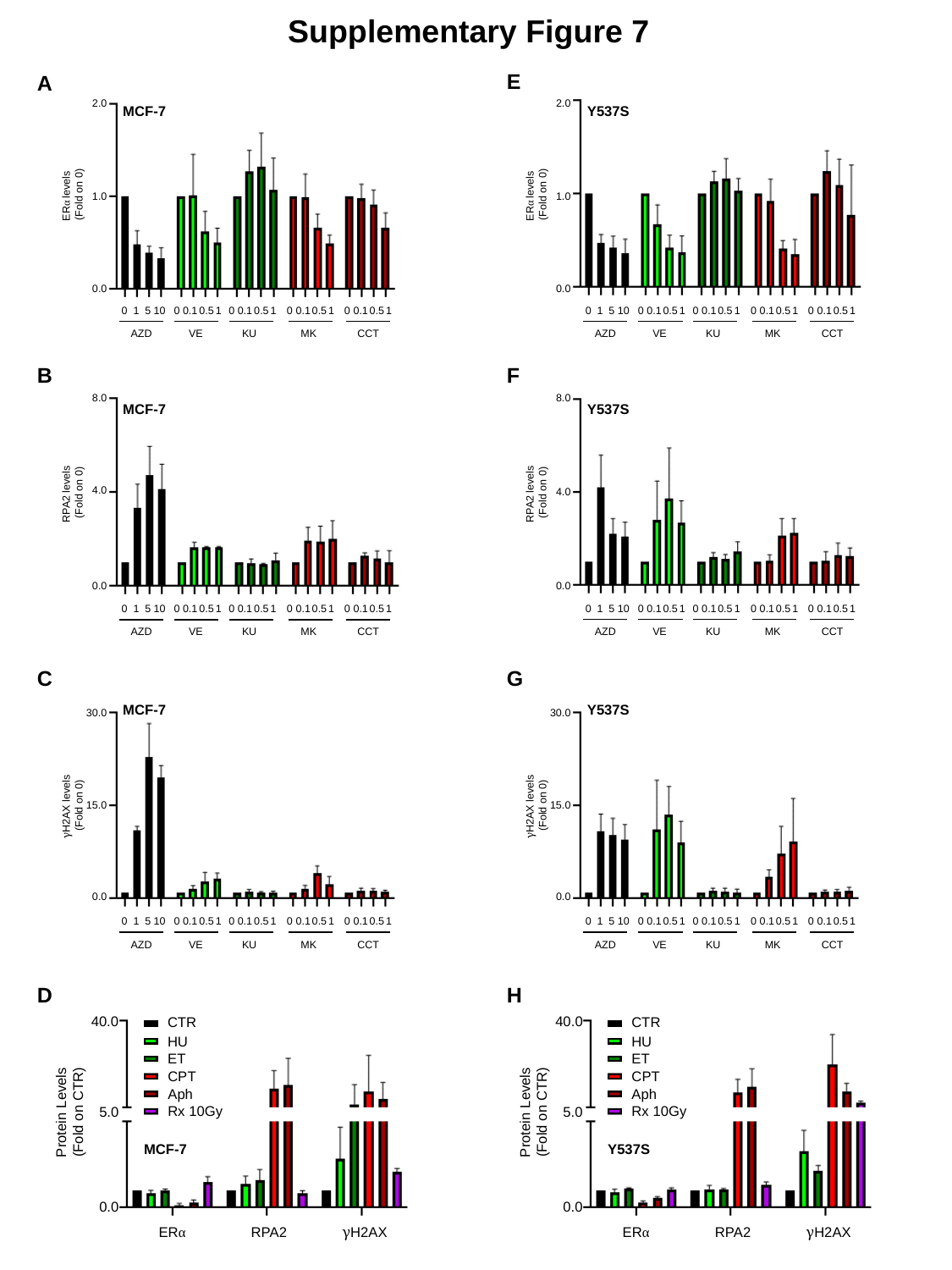

Supplementary Figure 7
E
A
2.0
MCF-7
ERα levels
(Fold on 0)
1.0
0.0
0
1
5
10
0
0.1
0.5
1
0
0.1
0.5
1
0
0.1
0.5
1
0
0.1
0.5
1
AZD
VE
KU
MK
CCT
2.0
Y537S
ERα levels
(Fold on 0)
1.0
0.0
0
1
5
10
0
0.1
0.5
1
0
0.1
0.5
1
0
0.1
0.5
1
0
0.1
0.5
1
AZD
VE
KU
MK
CCT
B
F
8.0
MCF-7
RPA2 levels
(Fold on 0)
4.0
0.0
0
1
5
10
0
0.1
0.5
1
0
0.1
0.5
1
0
0.1
0.5
1
0
0.1
0.5
1
AZD
VE
KU
MK
CCT
8.0
Y537S
RPA2 levels
(Fold on 0)
4.0
0.0
0
1
5
10
0
0.1
0.5
1
0
0.1
0.5
1
0
0.1
0.5
1
0
0.1
0.5
1
AZD
VE
KU
MK
CCT
C
G
MCF-7
30.0
γH2AX levels
(Fold on 0)
15.0
0.0
0
1
5
10
0
0.1
0.5
1
0
0.1
0.5
1
0
0.1
0.5
1
0
0.1
0.5
1
AZD
VE
KU
MK
CCT
Y537S
30.0
γH2AX levels
(Fold on 0)
15.0
0.0
0
1
5
10
0
0.1
0.5
1
0
0.1
0.5
1
0
0.1
0.5
1
0
0.1
0.5
1
AZD
VE
KU
MK
CCT
D
H
40.0
CTR
HU
ET
CPT
Aph
Protein Levels
(Fold on CTR)
Rx 10Gy
5.0
MCF-7
0.0
RPA2
γH2AX
ERα
40.0
CTR
HU
ET
CPT
Aph
Protein Levels
(Fold on CTR)
Rx 10Gy
5.0
Y537S
0.0
RPA2
γH2AX
ERα
